# Assessing the reporting quality of early phase dose-finding trial protocols: a methodological review

**DOI:** 10.1016/j.eclinm.2023.102020

**Published:** 2023-05-25

**Authors:** Guillermo Villacampa, Dhrusti Patel, Haiyan Zheng, Jessica McAleese, Jan Rekowski, Olga Solovyeva, Zhulin Yin, Christina Yap

**Affiliations:** a Clinical Trials and Statistics Unit at The Institute of Cancer Research (ICR-CTSU), United Kingdom; b MRC Biostatistics Unit, University of Cambridge, United Kingdom

**Keywords:** Dose-finding, Dose-escalation, Early phase trials, Protocols, SPIRIT, Oncology

## Abstract

**Background:**

The paradigm of early phase dose-finding trials has evolved in recent years. Innovative dose-finding designs and protocols which combine phases I and II are becoming more popular in health research. However, the quality of these trial protocols is unknown due to a lack of specific reporting guidelines. Here, we evaluated the reporting quality of dose-finding trial protocols.

**Methods:**

We conducted a cross-sectional study of oncology and non-oncology early phase dose-finding trial protocols posted on ClinicalTrials.gov in 2017–2023. A checklist of items comprising: 1) the original 33-items from the SPIRIT 2013 Statement and 2) additional items unique to dose-finding trials were used to assess reporting quality. The primary endpoint was the overall proportion of adequately reported items. This study was registered with PROSPERO (no: CRD42022314572).

**Finding:**

A total of 106 trial protocols were included in the study with the rule-based 3 + 3 being the most used trial design (39.6%). Eleven model-based and model-assisted designs were identified in oncology trials only (11/58, 19.0%). The overall proportion of adequately reported items was 65.1% (95%CI: 63.9–66.3%). However, the reporting quality of each individual item varied substantially (range 9.4%–100%). Oncology study protocols showed lower reporting quality than non-oncology. In the multivariable analysis, trials with larger sample sizes and industry funding were associated with higher proportions of adequately reported items (all p-values <0.05).

**Interpretation:**

The overall reporting quality of early phase dose-finding trial protocols is suboptimal (65.1%). There is a need for improved completeness and transparency in early phase dose-finding trial protocols to facilitate rigorous trial conduct, reproducibility and external review.

**Funding:**

None.

**Research in context**

**Evidence before this study**

Clearly written protocols, with provision of sufficient detail to help the reader understand the trial methodology and know what is pre-specified, are of paramount importance. The SPIRIT (Standard Protocol Items: Recommendation for Interventional Studies) 2013 Statement provides evidence-based recommendations for the minimum content of a clinical trial protocol. A PubMed search using the terms “*clinical trials*”, “*SPIRIT*”, “*dose-escalation*” and “*dose-finding*” (combined with the Boolean logic operation “*OR”/“AND*”) was performed in June 2022 to review the available evidence. Despite its broad applicability to many types of trials, SPIRIT 2013 and its extensions do not comprehensively cover the characteristics of early phase dose-finding trials and the quality of these protocols in an era with more adaptive designs is unknown.

**Added value of this study**

This study provides a comprehensive assessment of the reporting quality of early phase dose-finding trial protocols (from 2017 to 2023). The results identified i) the reporting quality of contemporary early phase dose-finding protocols, ii) the least frequently reported items, and iii) the type of trial protocols that need more surveillance actions. Additionally, this study assessed the prevalence of the most common trial designs used in phase I and I/II oncology and non-oncology trials.

**Implications of all the available evidence**

The findings can be used by the trial community to identify areas that have been poorly reported and/or require substantial improvement. Persistent and collaborative efforts from journals, editors, reviewers, regulators, funders and investigators are necessary to enhance the comprehensiveness and transparency of such protocols. This study highlights the need for an international consensus-driven protocol guidance for early phase dose-finding trials, and paves the way for creating recommendations of essential contents to be included in such protocols.

## Introduction

The paradigm of early phase dose-finding trials has dramatically evolved in recent years. Often termed “*dose-finding*” or “*dose escalation*” studies, early phase trials (phase I or I/II) are a critical step in clinical development. The primary objective of such trials is to assess the safety of an experimental treatment and finding recommended dosing regimen(s). They have historically been designed using classical rule-based approaches. However, as many innovative model-based designs for dose finding have been developed, more sophisticated designs have been implemented and protocols combining phases I and II are becoming more popular in health research.^1^^,^^2^

The current landscape of early phase clinical trials includes a broad variety of study designs. Typically involving several dose levels for evaluation, dose-finding trials feature the use of accumulating trial data for adaptive decision making. Dose assignment decisions are based on safety considerations, pharmacokinetic and pharmacodynamic data, biological markers or a combination of these parameters. Incomplete or unclear information on the design, conduct and analysis in dose-finding protocols would hinder their interpretability and reproducibility. This may impact on timely clinical development and can lead to inadequate reporting and erroneous conclusions on safety and efficacy data. In some circumstances, patient safety and ethics of the trial can be jeopardised, exposing patients to subtherapeutic or even harmful doses.^3^ Consequently, transparency and correct reporting of trial protocols are the key elements to improve clinical research.

The SPIRIT (Standard Protocol Items: Recommendation for Interventional Studies) 2013 Statement provides evidence-based recommendations for the minimum content of a clinical trial protocol.^4^ The SPIRIT Statement includes a 33-item checklist and is widely endorsed as an international standard for randomised control trial (RCT) protocols. Recent studies have shown that the reporting quality of published RCTs has improved since the SPIRIT statement.^5^ The SPIRIT 2013 and its extensions to date do not fully cover the features of early phase dose-finding trials^6^ and the quality of these protocols is unknown.

Considering this background, we performed a methodological study to assess the quality of contemporary dose-finding trial protocols. The aims of this study are 1) to determine the reporting quality of early phase dose-finding protocols, based broadly on the SPIRIT 2013 Statement with added items specific to early phase dose-finding trials, and 2) to determine whether the reporting quality differs by trial characteristics.

## Methods

### Study design

This methodological cross-sectional study evaluated the reporting quality of early phase dose-finding protocols. The study was registered in the international prospective register of systematic reviews PROSPERO (registration no: CRD42022314572).

### Included protocols

**Fig. 1:**
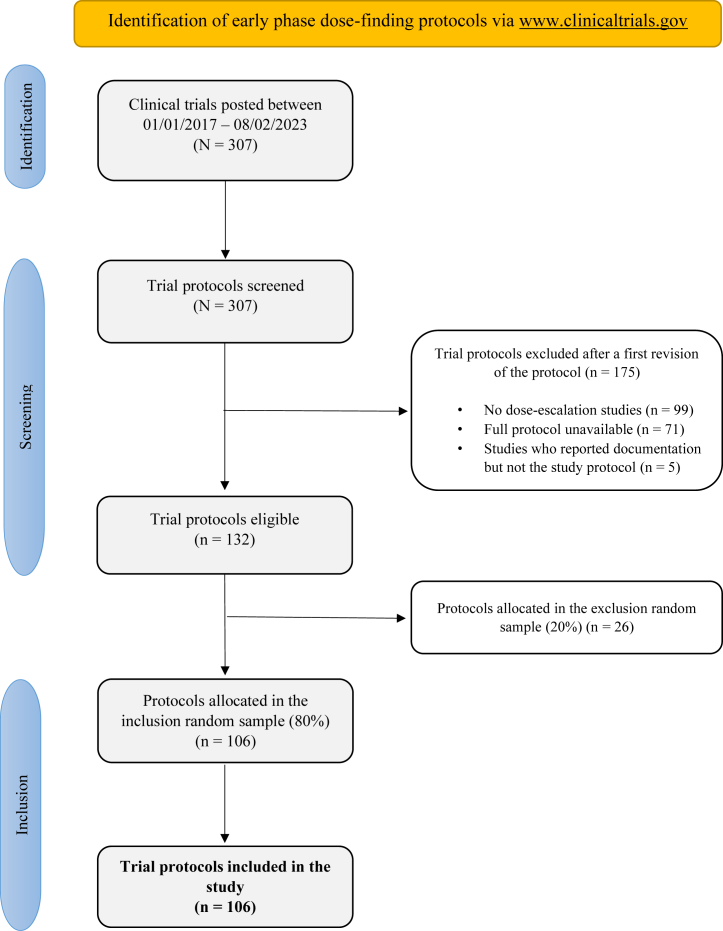
Flow diagram with the identification of early phase dose-finding protocols via www.clinicaltrials.gov.

To be included in this study, protocols had to meet the following criteria: 1) phase I or phase I/II trials that include a dose (de-)escalation component, 2) treated living humans, 3) had their full protocols in English, and 4) posted between 01/01/2017 and 08/02/2023 in ClinicalTrials.gov. No restrictions were applied to limit types of intervention or setting. To identify the potential full protocols, an electronic systematic search was performed via ClinicalTrials.gov. Details of the systematic search can be found in Fig. 1 and Supplementary materials. Examination of potentially eligible protocols was performed independently by two authors (GV and DP) based on titles and a first screen of the protocol; potential disagreements were resolved by a third author (CY). A random sample of 80% of all eligible trial protocols were selected for complete revision and 106 full protocols were finally included in the study.

### Variables

A standardised Excel spreadsheet was created to extract data from the included protocols. The following variables were collected for all protocols if available: Clinicaltrials.gov identifier, study title, first author (or principal investigator), study sample size (of the dose-escalation component only), area of research, protocol year, research funding, multi-centre or single-centre study and type of the dose-escalation design.

Additionally, to evaluate the reporting quality of the protocols, a checklist of items was created as a combination of 1) original items from SPIRIT 2013 and modified items tailored to dose-finding trials and 2) additional items unique to dose-finding trials considered useful by the review team. The complete list of all 68 items can be found in Supplementary Table S1. The selected items were evaluated in all included studies. Each item was assessed as "*Yes*" or "*No*". For some items, there was the option to select "*Not applicable*" and these items were not evaluated. For composite items, the option "*Partially*" was also available (examples of partially reported items can be found in the Supplementary materials).

### Assessment strategy

All included protocols were randomly allocated to at least one member of the study team. Thirteen protocols (12.3%) were reviewed by more than one member to check for discrepancies or potentially spurious data. To standardise the assessment criteria, two roundtable discussions were held during the evaluation period. The first roundtable took place among all authors to discuss the first 8 double-reviewed protocols, where each protocol had been independently reviewed by two authors beforehand. The second roundtable took place when 24 protocols had been assessed in order to review and discuss discrepancies. As a result of these meetings, an internal document was created with examples of good reporting for each item to provide guidance in the subsequent individual protocol assessment (more information regarding the examples and the assessment strategy can be found in Supplementary materials).

### Outcomes

The primary endpoint for this study was the overall proportion of items adequately reported in all evaluated trial protocols (SPIRIT items and items unique to dose-finding trials). Explicitly, this metric was defined as the number of adequately reported items in all evaluated protocols divided by the total number of applicable items. High proportions would suggest high reproducibility and quality of dose-finding trial protocols. The study also had the following secondary endpoints: 1) percentage of protocols that had reported each item adequately, 2) factors associated with the quality of the protocol and 3) prevalence of the most common statistical designs used oncology and non-oncology in phase I or phase I/II trials.

### Statistical methods

The overall proportion of adequately reported items and the percentage of protocols that had been adequately reported per individual item were reported, alongside its corresponding 95% confidence interval (CI) calculated using the Clopper-Pearson method. Items evaluated as “*Yes*” or “*Partially*” were combined as ‘*adequately reported*’ to facilitate result interpretation. Univariable linear regression models were used to study the association between factors of interest and the overall proportion of adequately reported items. A multivariable linear model was evaluated to further identify attributes of the quality of protocol, where the least absolute shrinkage and selection operator (LASSO) regression was used for variable selection. No data imputation was performed and a significance level of 0.05 was set for a two-sided test. All analyses were performed using R statistical software version 4.1.2.

### Role of the funding source

No funding was received for this work.

## Results

A total of 106 trial protocols were included in the study (Fig. 1) and a summary of the trial characteristics can be found in Table 1. Briefly, 58 (54.7%) were oncology trials, 71 (67.0%) were industry funded, 33 (31.1%) were single-centre and the median sample size for the dose-finding part was 36 patients (Q1-Q3, 20–64). Thirty-five (33.0%) of the included studies used randomisation within the dose-escalation part, but the percentage of randomised trials was substantially higher in non-oncology compared to oncology trials (66.7% versus 5.2%, respectively).

**Table 1:** Characteristics of included trial protocols. Overall and by field (oncology and non-oncology).

	Overall (n = 106)	Oncology trials (n = 58)	Non-oncology trials (n = 48)
Sample size (median and IQR)	36 (20–64)	30 (18–45)	48 (29–72)
**Center,** n (%)			
Multi-center	54 (51.0)	34 (58.6)	20 (41.7)
Single-center	33 (31.1)	14 (24.1)	19 (39.6)
Unclear	19 (17.9)	10 (17.2)	9 (18.8)
**Funding**, n (%)			
Industry	71 (67.0)	41 (70.7)	30 (62.5)
Non-industry	32 (30.2)	16 (27.6)	16 (33.3)
Unclear	3 (2.8)	1 (1.7)	2 (4.2)
**Field**, n (%)			
Oncology	58 (54.7)	58 (100)	0 (0)
Non-oncology	48 (45.3)	0 (0)	48 (100)
**Randomisation**, n (%)			
No	71 (67.0)	55 (94.8)	16 (33.3)
Yes	35 (33.0)	3 (5.2)	32 (66.7)
**Trial design**, n (%)			
Rule-based	55 (51.9)	42 (72.4)	13 (27.1)
SAD and MAD/SAD/MAD	20 (18.9)	0 (0)	20 (41.7)
Model-assisted	7 (6.6)	7 (12.1)	0 (0)
Model-based	4 (3.7)	4 (6.9)	0 (0)
Others	20 (18.9)	5 (8.6)	15 (31.2)

IQR: interquartile range; SAD and MAD: single ascending dose and multiple ascending dose.

**Fig. 2:**
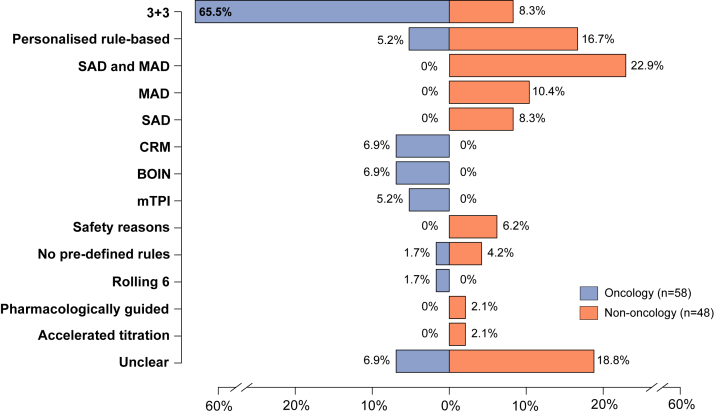
Trial designs used in the oncology and non-oncology dose-finding studies. SAD and MAD: single ascending doses and multiple ascending doses; CRM: continual reassessment method; BOIN: Bayesian optimal interval; mTPI: modified toxicity probability interval.

Overall, the most used design was the rule-based 3 + 3 design (42/106, 39.6%), followed by personalised rule-based designs (11/106, 10.4%) and Single/Multiple Ascending Dose (SAD and MAD) designs (11/106, 10.4%). There were differences in the types of designs used in the oncology and non-oncology setting (Fig. 2). The SAD, MAD or SAD and MAD designs were only found in non-oncology trials and were the most common designs in that setting (20/48, 41.7%). In oncology, the 3 + 3 was used in most of the trials (38/58, 65.5%), whereas the prevalence of this design decreased to 8.3% in non-oncology trials. Eleven model-based and model-assisted approaches were found only in oncology trials (4 continual reassessment method [CRM], 4 Bayesian Optimal Interval [BOIN] and 3 modified toxicity probability interval (mTPI) designs).

### Proportion of adequately reported items

**Fig. 3:**
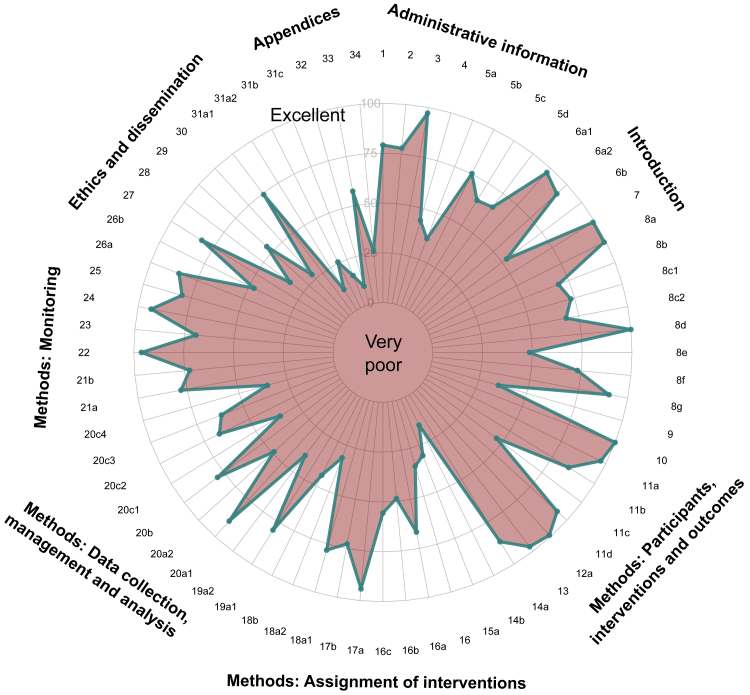
Radar chart to show the adequately reported percentage for each individual item (the closer the percentage is to 100%, the better the reporting quality). Legend: 1. Title; 2. Trial registration; 3. Protocol version; 4. Funding; 5a-5b-5c-5d. Roles and responsibilities; 6a1-6a2-6b background and rationale; 7. Objectives; 8a-8b-8c1-8c2-8d-8e-8f-8g-9 Trial design; 10. Eligibility criteria; 11a-11b-11c-11d Interventions; 12a. Outcomes; 13. Participant timeline; 14a-14b. Sample size; 15a Recruitment; 16. Randomization; 16a-16b-16c; 17a-17b Blinding; 18a1-18a2-18b Data collection methods; 19a1-19a2 Data management; 20a1-20a2-20b-20c1-20c2-20c3-20c4 Statistical methods; 21a-21b Data monitoring; 22 Harms; 23 Auditing; 24. Research ethics approval; 25. Protocol amendments; 26a-26b Consent or assent; 27. Confidentially; 28 Declaration of interest; 29. Access to data; 30. Ancillary and post-trial care; 31a1-31a2-31b-31c. Dissemination policy; 32. Informed consent materials; 33. Biological specimens; 34. Dose transition pathways.

In the 106 trial protocols included in the study, the overall proportion of adequately reported items was 65.1% (95% CI: 63.9–66.3%). The percentage of protocols with more than 70% of items reported adequately was 22.6% (n = 24) and only 1.9% (n = 2) reported more than 80% adequately. Interestingly, the reporting quality of each individual item varied substantially: the percentage of protocols that reported a specific item ranged from 9.4% to 100% (Fig. 3). On the one hand, the best reported items were the trial objectives (item 7), trial design: planned cohort size (item 8) and eligibility criteria (item 10), all being reported in 100% of protocols. On the other hand, informed consent materials (item 32), dissemination policy (item 31a) and sample size: operating characteristics (item 14 b) were the least frequently stated items with a percentage of 9.4%, 12.0% and 15.4%, respectively. The exact percentage of protocols that adequately reported each item can be found in Supplementary Table S2.

### Factors associated to quality of protocol

**Fig. 4:**
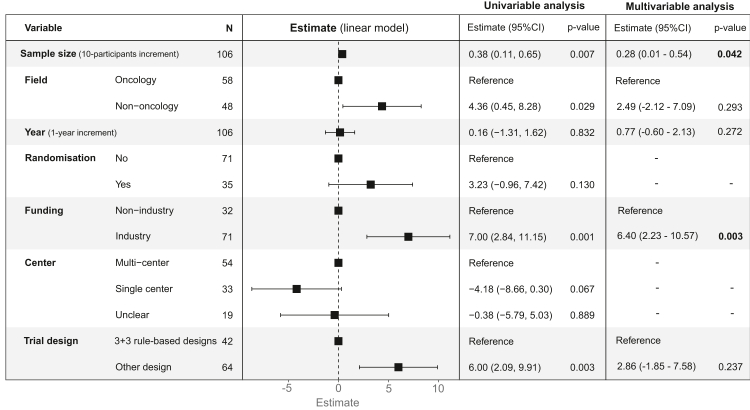
Univariable and multivariable linear regression model to evaluate the association between trial characteristics and the percentage of adequately reported items. LASSO regression was used to select factors for the multivariable regression analysis. 95% confidence interval.

Fig. 4 shows the association between trial characteristics and the quality of the protocol (percentage of adequately reported items). In the univariable analysis, studies with larger sample sizes, non-oncology, industry funding and studies with a design different than the 3 + 3 for dose-finding were associated with higher proportions of adequately reported items. In the multivariable regression analysis, study sample size (effect size per 10-participants increment: 0.28, 95% CI: 0.01–0.54) and funding source (effect size: 6.40; 95% CI: 2.23–10.57) were the only factors that maintained their independent statistical significance.

**Fig. 5:**
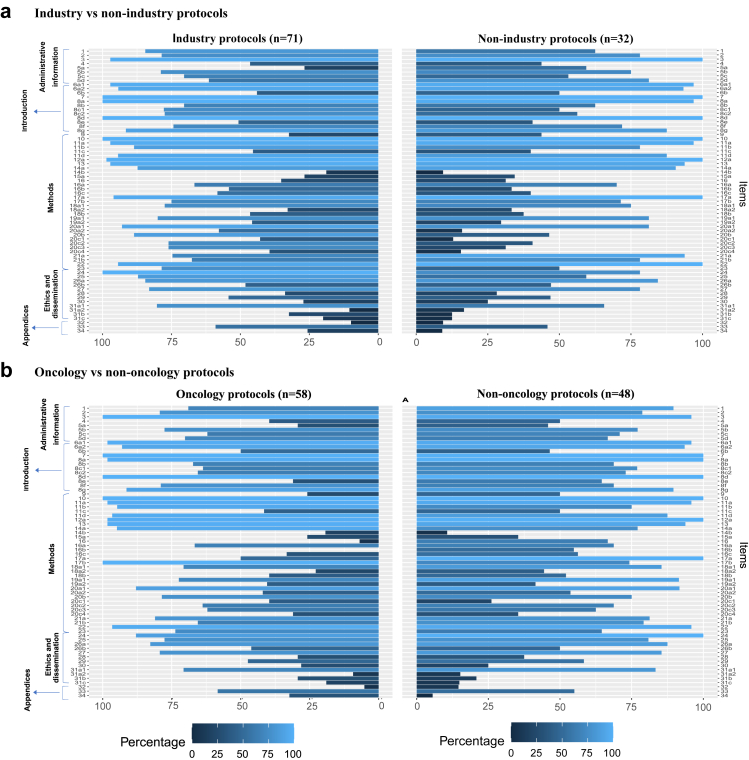
Horizontal mirror bar chart to evaluate reporting quality differences regarding trial characteristics in all individual items. (a) Comparison between industry (n = 71) and non-industry-funded (n = 32) trials. (b) Comparison between oncology (n = 58) and non-oncology (n = 48) trials.

To better characterise the potential differences resulting from funding source or disease area, we compared the reporting quality of each item by i) industry versus non-industry trials and ii) oncology versus non-oncology trials (Fig. 5). Overall, industry-funded trials reported the statistical methods (all sub-items of item 20) and the audit plan for the trial (item 23) more often than non-industry trials. Non-oncology trial protocols showed better reporting quality in the methods section as well as in ethnics and dissemination.

## Discussion

The classical paradigm of drug development in health research consisting of three separate phases (I-III) has left its place to a more dynamic paradigm with less barriers between phases. In oncology, the current early-to-late transition, the strategy to move from a promising phase I/II trial directly to a confirmatory phase III trials, highlights the importance of correctly selecting treatment dose level to maximise the chances of a successful further development. Moreover, specific examples of drugs that were evaluated using a potential suboptimal dose have reinforced the necessity to design efficient, robust and replicable early phase trials.^7^^,^^8^

Although a well-reported dose-finding trial protocol is vital even for simple designs, as the trial designs evolve and get more complex, they are technically more complex to implement than conventional designs. This is especially so to assist in the conduct of trials that require the use of sophisticated and adaptive methods to achieve various objectives simultaneously. The SPIRIT 2013 Statement emerged as a guide to improve the reporting quality of trial protocols. However, the recommendations provided by SPIRIT were created in an era with less personalised and adaptive trial designs and the current quality of early phase trial protocols is unknown due to a lack of specific reporting guidelines.^4^^,^^5^ Currently, the DEFINE (DosE-FIndiNg Extensions) project is developing extensions of the SPIRIT 2013 and CONSORT 2010 statements for early phase dose-finding clinical trials.^6^^,^^9^

This methodological review evaluated early phase dose-finding protocols from 2017 to 2023 showing a suboptimal quality in the reporting (65.1% of the items were reported adequately). Results from this study are consistent with those previously reported in RCTs trials (56.7%),^5^ although the checklist of items and the timeframe were not the same and direct comparison should be avoided. In the assessment of each individual item, the proportion varied substantially, meaning that some items were reported systematically better than others. Information related to the eligibility criteria, objectives, protocol version and primary-secondary outcomes were correctly reported in most trial protocols (>95%). Contrarily, sections on ethics and dissemination were not frequently outlined. In particular, items related to the dissemination policy, consent material information and ancillary and post-trial care presented an especially poor reporting quality (<30%). Interestingly, the importance of improving transparency and consent information in phase I oncology trials has been recently discussed.^10^ Our study is in line with these discussions showing that informed consent documentation was among the worst reporting items.

The items used for evaluating the reporting quality of early phase dose-finding protocols were based broadly on SPIRIT 2013, with additional protocol-related items drawn from the comparison guidance for trial reports of early phase dose-finding trials (in development),^9^ literature review of reporting guidance, expert opinions and unpublished literature including regulatory and industry advice documents as detailed elsewhere.^6^ Some of the expanded items can be considered sub-items of the original SPIRIT item. For instance, the original SPIRIT 2013 item 8 on trial design has been expanded to 8 sub-items capturing multifaceted features of early phase dose-finding trial design such as rationale for starting dose, cohort size, and pre-planned guidance for trial (dose) adaptations.

Subgroup analysis helped to better identify the type of trial protocols, according to study characteristics, that need more surveillance actions. Non-industry studies with a relatively small sample size presented the lowest reporting quality, and conversely, industry-funded studies, with the use of novel designs and a generally larger sample size, presented a higher reporting quality. In particular, the better reporting quality of industry-funded studies in some sections (e.g., statistical methods and audit plan) could be explained by the fact that these protocols are more likely to be reviewed by regulatory agencies. Non-oncology protocols also showed better reporting quality than oncology protocols. However, this should be interpreted alongside the fact that types of early phase dose-finding designs are notably different across therapeutic areas. Specifically, most of the non-oncology dose-finding trial were randomised studies (66.7%), including a median of 48 patients in the dose-finding part. Whereas randomisation was rarely used in oncology trials (5.2%) and the number of patients evaluable per dose levels was smaller.

Although model-based and model-assisted designs had shown better operating characteristics in the dose selection process than rule-based designs,^11^, ^12^, ^13^ we found limited use of these designs in the overall spectrum of early phase trials (10.3%). In oncology trials this percentage increased to 19% (CRM, BOIN and mTPI designs), which is a marked increment compared to the 8.6% showed in previous review in 2014–2019.^14^ The classical 3 + 3 ruled-based design was still by far the most used design in oncology trials, while the integrated SAD and MAD designs were the most used in the non-oncology setting. Those results suggest that future development of efficient dose-finding designs needs to be made easy to implement to facilitate the uptake in different fields of health research.^15^

This study has some limitations. First, subjectivity on the part of the reviewers could occur when the reporting was unclear to draw any conclusions, even though an assessment strategy was prepared to minimise the risk. And second, the study was based on a random sample of 80% of eligible protocols. Though it is considered sufficiently large to provide an overall assessment of reporting quality of dose-finding protocols, the number of protocols with some specific trial characteristics could be too small to extract robust conclusions. Strengths include being the first study to comprehensively assess the reporting quality of early phase dose-finding trial protocols, and its wide scope covering both oncology and non-oncology therapeutic areas. Additionally, we have considered a list of additional items that address design features specific to dose-finding procedures to complement the SPIRIT checklist.

In conclusion, the current reporting quality of early phase dose-finding trial protocols is suboptimal. These results could be used by the trial community to identify areas that have been poorly reported and require substantial improvement. Additionally, it paves the way for creating a consensus-driven guidance of the minimum essential content to be included in early phase dose-finding trial protocols. This guidance could improve reporting practices and transparency to facilitate the rigorous conduct of future trials.

## Contributors

Specific author contributions are as follows. Guillermo Villacampa: Methodology, Validation, Investigation, Data curation, Formal analysis, Visualization, Writing - original draft, Writing - Review & Editing, Project administration. Dhrusti Patel: Methodology, Validation, Investigation, Data curation, Writing - original draft, Writing - Review & Editing, Project administration. Haiyan Zheng: Methodology, Investigation, Data curation, Writing - original draft, Writing - Review & Editing. Jessica McAleese: Investigation, Data curation, Formal analysis, Visualization, Writing - review & editing. Jan Rekowski: Methodology, Investigation, Data curation, Methodology, Writing - review & editing. Olga Solovyeva: Methodology, Investigation, Data curation, Methodology, Writing - review & editing. Zhulin Yin: Methodology, Investigation, Data curation, Methodology, Writing - review & editing. Christina Yap: Conceptualization, Methodology, Investigation, Data curation, Writing - original draft, Writing - Review & Editing, Project administration, Supervision.

The findings, interpretations and conclusions expressed in this paper are entirely those of the authors. The funding source did not have a role in the writing or decision to submit for publication. All authors have full access to the full data in the study and accept responsibility to submit for publication.

## Data sharing statement

All reasonable requests for data sharing will be considered and should be emailed to Christina Yap (christina.yap@icr.ac.uk).

## Declaration of interests

Guillermo Villacampa has received a speaker's fee from MSD, Pfizer, GSK and Pierre Fabre, has held an advisory role with AstraZeneca and received consultant fees from Reveal Genomics. All other authors declare no competing interests.
