# Supplementary material for: Assessing the reporting quality of early phase dose-finding trial protocols: a methodological review
Source: eClinicalMedicine. 2023 May 25;60:102020. doi: 10.1016/j.eclinm.2023.102020 (PMC10227378; doi:10.1016/j.eclinm.2023.102020)
Supplement: Supplementary material [file mmc1.docx]

**SUPPLEMENTARY MATERIALS**

[**Search Strategy** 2](#_Toc129087293)

[Selection process 2](#_Toc129087294)

[**Supplementary Tables** 3](#_Toc129087296)

[Supplementary Table 1: Data items reviewed for in early phase dose-finding trial protocols 3](#_Toc129087297)

[Supplementary Table 2: Percentage of protocols that adequately reported each item 10](#_Toc129087298)

[**Supplementary Figures** 15](#_Toc129087299)

[**S**upplementary Figure 1: Overview of the assessment strategy 15](#_Toc129087300)

[**Supplementary Examples Information** 16](#_Toc129087299)

Illustrative examples of adequately and partially reported items in the evaluated dose-findings trial protocol [16](#_Toc129087300)

Handbook with examples of adequately reported items  [18](#_Toc129087300)

# **Search strategy**

## **Selection process**

Search criteria used to obtain protocols from [www.clinicaltrials.gov](http://www.clinicaltrials.gov)

Search was originally conducted on 21^th^ of March 2022 (from 01/01/2017 to 01/01/2022) and later updated on 8^th^ of February 2023 (from 01/01/2022 to 08/02/2023).

Advanced search:

- Other terms: “Dose escalation”
- Study type: Interventional studies (Clinical trials)
- Study results: All studies
- Additional criteria – Phase:
  - Early phase 1
  - Phase 1
  - Phase 2
- Additional criteria – study documents:
  - Study protocols
- First posted from 01/01/2017 to 08/02/2023

# **Supplementary Tables**

## **Supplementary Table 1**: Data items reviewed in early phase dose-finding trial protocols

| **Category** | **Data Items** | **SPIRIT 2013 Item No.** | **SPIRIT 2013 Item Description** | **Review Item No.** | **Description Of Item Reviewed**  (M: modification from SPIRIT 2013, N: new dose-finding specific item) | **Rating Options** |
| --- | --- | --- | --- | --- | --- | --- |
| Administrative Information | Title | 1 | Descriptive title identifying the study design, population, interventions, and, if applicable, trial acronym. | 1 | [M] Descriptive title identifying the study design (e.g. first-in-human, early phase dose-finding, dose escalation/de-escalation, single-/multiple-ascending dose, phase I, (seamless) phase I/II or dose-titration), population, interventions, and, if applicable, randomisation and/or trial acronym | Yes/No |
|  | Trial Registration | 2a | Registry – Trial identifier and registry name. If not yet registered, name of intended registry. | 2 | Unchanged | Yes/No |
|  |  | 2b | Data set – All items from the World Health Organization Trial Registration Data Set. |  | Not reviewed |  |
|  | Protocol Version | 3 | Date and version identifier. | 3 | Unchanged | Yes/No |
|  | Funding | 4 | Sources and types of financial, material, and other support. | 4 | Unchanged | Yes/No |
|  | Roles And Responsibilities | 5a | Contributorship - Names, affiliations, and roles of protocol contributors. | 5a | Unchanged | Yes/No |
|  |  | 5b | Sponsor contact information – Name and contact information for the trial sponsor. | 5b | Unchanged | Yes/No |
|  |  | 5c | Sponsor and funder – Role of study sponsor and funders, if any, in study design; collection, management, analysis, and interpretation of data; writing of the report; and the decision to submit the report for publication, including whether they will have ultimate authority over any of these activities. | 5c | Unchanged | Yes/No |
|  |  | 5d | Committees - Composition, roles, and responsibilities of the coordinating centre, steering committee, endpoint adjudication committee, data management team, and other individuals or groups overseeing the trial, if applicable | 5d | Unchanged | Yes/ No/ Partially/ Not applicable |
| Introduction | Background And Rationale | 6a | Description of research question and justification for undertaking the trial, including summary of relevant studies (published and unpublished) examining benefits and harms for each intervention. | 6a.1 | [M] Description of research question and justification for undertaking the trial, including: summary of findings from nonclinical (e.g. in vitro, in vivo and/or in silico studies) that have potential clinical significance | Yes/No |
|  |  |  |  | 6a.2 | [N] If applicable, summary of pre-clinical/non-clinical research | Yes/No/ Not applicable |
|  |  | 6b | Explanation for choice of comparators. | 6b | Unchanged | Yes/No/ Not applicable |
|  | Objectives | 7 | Specific objectives or hypotheses. | 7 | [M] Specific objectives or hypotheses; defining clearly what the key objectives are (e.g., primary and secondary objectives that encompasses: safety, toxicity, activity/efficacy, pharmacokinetics, pharmacodynamics, feasibility assessment, or some combination) | Yes/ No/ Partially |
|  | Trial Design | 8 | Description of trial design including type of trial (e.g., parallel group, crossover, factorial, single group), allocation ratio, and framework (e.g., superiority, equivalence, non-inferiority, exploratory). | 8a | [M] Description of type and design of trial:  • Phase  • Type of trial (e.g. open-label, double-blinded, placebo-controlled, dose-escalation, expansion cohort, intra-patient dose escalation)  • Framework (if applicable, Bayesian or frequentist hypothesis testing),  • Number of study groups/arms  with rationale. | Yes/ No/ Partially |
|  |  |  |  | 8b | [N] Is the trial design schema provided? | Yes/ No/ |
|  |  |  |  | 8c.1 | [N] Where applicable, details regarding the statistical methodology underpinning the trial, including the choice of the number of parameters in the model if applicable, its empirical form and all formulae | Yes/No/ Not applicable |
|  |  |  |  | 8c.2 | [N] Rationale for starting dose | Yes/ No/ Partially |
|  |  |  |  | 8d | [N] Planned cohort size(s) (fixed or flexible) | Yes/ No |
|  |  |  |  | 8e | [N] Sequence and interval between dosing of participants (e.g. sentinel/staggered dosing at each untested dose level or between dose levels). | Yes/No/ Not applicable |
|  |  |  |  | 8f | [N] Pre-planned guidance/rules for trial adaptations (e.g. dose [de-]escalation strategy), when they will occur and the information (e.g. toxicity, response, PK/PD, either singularly or in combination) used to make the adaptations. | Yes/ No |
|  |  |  |  | 8g | [N] Stopping criteria for treatment group or trial (e.g. progression to the next part of the trial, early termination of a group or the trial due to e.g. safety, futility or efficacy) and consequences. | Yes/ No |
| Methods: Participants, Interventions, Outcomes | Study Setting | 9 | Description of study settings (e.g., community clinic, academic hospital) and list of countries where data will be collected. Reference to where list of study sites can be obtained. | 9 | Unchanged | Yes/ No/ Partially |
|  | Eligibility Criteria | 10 | Inclusion and exclusion criteria for participants. If applicable, eligibility criteria for study centres and individuals who will perform the interventions (e.g., surgeons, psychotherapists). | 10 | [M] Inclusion and exclusion criteria for participants. | Yes/ No |
|  | Interventions | 11a | Interventions for each group with sufficient detail to allow replication, including how and when they will be administered. | 11a | [M] The interventions for each dose level within each treatment group with sufficient details to allow replication, including administration, route, and schedule showing how and when they will be administered. | Yes/ No/ Partially |
|  |  | 11b | Criteria for discontinuing or modifying allocated interventions for a given trial participant (e.g., drug dose change in response to harms, participant request, or improving/worsening disease). | 11b | [M] Criteria for discontinuing, dose modifications and dosing delays of allocated interventions for a given trial participant (e.g. drug dose change in response to harms, participant request, or improving /worsening disease) | Yes/ No |
|  |  | 11c | Strategies to improve adherence to intervention protocols, and any procedures for monitoring adherence (e.g., drug tablet return; laboratory tests). | 11c | Unchanged | Yes/ No/ Not applicable |
|  |  | 11d | Relevant concomitant care and interventions that are permitted or prohibited during the trial. | 11d | Unchanged | Yes/ No |
|  | Outcomes | 12 | Primary, secondary, and other outcomes, including the specific measurement variable (e.g., systolic blood pressure), analysis metric (e.g., change from baseline, final value, time to event), method of aggregation (e.g., median, proportion), and time point for each outcome. Explanation of the clinical relevance of chosen efficacy and harm outcomes is strongly recommended. | 12 | [M] Primary, secondary and other outcome measures, including the specific measurement variable (eg, dose limiting toxicity based on drug-related adverse events), analysis metric (eg, change from baseline, final value, time to event), method of aggregation (eg, median, proportion), and time point for each outcome. Explanation of the clinical relevance of chosen efficacy and harm outcomes | Yes/ No/ Partially |
|  | Participant Timeline | 13 | Time schedule of enrolment, interventions (including any run-ins and washouts), assessments, and visits for participants. A schematic diagram is highly recommended. | 13 | [M] Time schedule of enrolment, interventions (including any run-ins and washouts), assessments, and visits for participants (including in-house stay or out-patient follow-up period where applicable). A schematic diagram is highly recommended. | Yes/ No |
|  | Sample Size | 14 | Estimated number of participants needed to achieve study objectives and how it was determined, including clinical and statistical assumptions supporting any sample size calculations. | 14a.1 | [M] Estimated number of participants (minimum/lower bound, maximum or expected range) needed to address study objectives and how it was determined, including clinical and statistical assumptions | Yes/ No |
|  |  |  |  | 14a.2 | [N] Were simulations/operating characteristics provided? | Yes/ No |
|  | Recruitment | 15 | Strategies for achieving adequate participant enrolment to reach target sample size. | 15 | [M] Strategies for achieving adequate participant enrolment to reach target sample size. Method of recruitment (e.g., referral, self-selection), including the sampling method if a systematic sampling plan to be implemented | Yes/ No |
| Methods: Assignment Of Interventions (For Controlled Trials) | Allocation | 16a | Sequence Generation:  Method of generating the allocation sequence (eg, computer-generated random numbers), and list of any factors for stratification. To reduce predictability of a random sequence, details of any planned restriction (eg, blocking) should be provided in a separate document that is unavailable to those who enrol participants or assign interventions | 16 | Is the trial randomised? | Yes/ No |
|  |  |  |  | 16a | Unchanged | Yes/ No/ Partially/ Not applicable |
|  |  | 16b | Concealment mechanism: Mechanism of implementing the allocation sequence (eg, central telephone; sequentially numbered, opaque, sealed envelopes), describing any steps to conceal the sequence until interventions are assigned | 16b | Unchanged | Yes/ No/ Partially/ Not applicable |
|  |  | 16c | Implementation: Who will generate the allocation sequence, who will enrol participants, and who will assign participants to interventions. | 16c | [M] Logistics: Who will generate the allocation sequence, who will enrol participants, and who will assign participants to interventions. Inclusion of aspects employed to help minimize potential bias induced due to non-randomization if applicable (e.g., matching) | Yes/ No/ Partially/ Not applicable |
|  | Blinding (Masking) | 17a | Who will be blinded after assignment to interventions (eg, trial participants, care providers, outcome assessors, data analysts), and how | 17a | Unchanged | Yes/ No/ Partially/ Not applicable |
|  |  | 17b | If blinded, circumstances under which unblinding is permissible, and procedure for revealing a participant’s allocated intervention during the trial. | 17b | Unchanged | Yes/ No/ Partially/ Not applicable |
| Methods: Data Collection, Management, Analysis | Data Collection Methods | 18a | Plans for assessment and collection of outcome, baseline, and other trial data, including any related processes to promote data quality (e.g., duplicate measurements, training of assessors) and a description of study instruments (e.g., questionnaires, laboratory tests) along with their reliability and validity, if known. Reference to where data collection forms can be found, if not in the protocol. | 18a.1 | [M] Plans for assessment and collection of outcome, baseline, and other trial data, including any related processes to promote data quality (eg, duplicate measurements, training of assessors) and a description of study instruments (eg, questionnaires, laboratory tests) along with their reliability and validity, if known. | Yes/ No |
|  |  |  |  | 18a.2 | [M] Reference to where data collection forms can be found, if not in the protocol | Yes/ No/ Not applicable |
|  |  | 18b | Plans to promote participant retention and complete follow-up, including list of any outcome data to be collected for participants who discontinue or deviate from intervention protocols | 18b | Unchanged | Yes/ No |
|  | Data Management | 19 | Plans for data entry, coding, security, and storage, including any related processes to promote data quality (e.g., double data entry; range checks for data values). Reference to where details of data management procedures can be found, if not in the protocol. | 19a.1 | [M] Plans for data entry, coding, security, and storage, including any related processes to promote data quality (eg, double data entry; range checks for data values). | Yes/ No |
|  |  |  |  | 19a.2 | [M] Reference to where details of data management procedures can be found, if not in the protocol.  Where applicable, specify if the plans in the initial dose-finding component are different from subsequent stages of the trial. | Yes/ No/ Not applicable |
|  | Statistical methods | 20a | Outcomes – Statistical methods for analysing primary and secondary outcomes. Reference to where other details of the statistical analysis plan can be found, if not in the protocol. | 20a.1 | [M] Statistical methods used for primary and secondary outcomes, and any other outcomes used to make pre-planned adaptations (e.g., determining the next participant’s dose level). | Yes/ No/ Partially |
|  |  |  |  | 20a.2 | [M] Reference to where other details of the statistical analysis plan can be found, if not in the protocol. | Yes/ No/ Not applicable |
|  |  | 20b | Additional analyses – Methods for any additional analyses (e.g., subgroup and adjusted analyses). | 20b | [M] Methods for any additional analyses (e.g. subgroup and adjusted analyses, PK/PD, biomarker correlative analyses) or where they can be found, if not in the protocol. | Yes/ No/ Not applicable |
|  |  | 20c | Analyses population and missing data – Definition of analysis population relating to protocol non-adherence (e.g., as randomised analysis), and any statistical methods to handle missing data (e.g., multiple imputation). | 20c.1 | [N] Definition of dose-escalation analysis population | Yes/ No |
|  |  |  |  | 20c.2 | [N] Definition of safety population | Yes/ No |
|  |  |  |  | 20c.3 | [N] Definition of other key outcomes population | Yes/ No |
|  |  |  |  | 20c.4 | [N] Statistical methods to handle missing data | Yes/ No |
| Methods: Monitoring | Data monitoring | 21a | Formal committee – Composition of data monitoring committee (DMC); summary of its role and reporting structure; statement of whether it is independent from the sponsor and competing interests; and reference to where further details about its charter can be found, if not in the protocol. Alternatively, an explanation of why a DMC is not needed. | 21a | [M] Composition of any decision-making group or safety review committee or data (safety) monitoring committee (DMC); summary of its role and reporting structure; statement of whether it is independent from the sponsor, funder or trials team and competing interests; and reference to where further details about its charter can be found, if not in the protocol. Alternatively, an explanation of why such a committee is not needed. | Yes/ No/ Partially |
|  |  | 21b | Interim analysis – Description of any interim analyses and stopping guidelines, including who will have access to these interim results and make the final decision to terminate the trial. | 21b | [M] Description of the plans for any interim data review (including timing and data to be used for decision-making) and interim statistical analyses (e.g., safety/toxicity, determination of the next participant’s dose, early stopping of dose level, treatment group or trial), including who will  - have access to these interim results and  - make the dose decisions  - make the final decision to terminate the trial | Yes/ No/ Partially |
|  | Harms | 22 | Plans for collecting, assessing, reporting, and managing solicited and spontaneously reported adverse events and other unintended effects of trial interventions or trial conduct. | 22 | [M] Plans for collecting, assessing, reporting, and managing solicited and spontaneously reported adverse events and other unintended effects of trial interventions (e.g. prior to any planned next dosing) or trial conduct | Yes/ No/ Partially |
|  | Auditing | 23 | Frequency and procedures for auditing trial conduct, if any, and whether the process will be independent from investigators and the sponsor. | 23 | [M] Frequency and procedures for auditing trial conduct (including specifying any difference moving from e.g. FIH/phase I to dose expansion or phase II), if any, and whether the process will be independent from investigators and the sponsor. | Yes/ No |
| Ethics And Dissemination | Research Ethics Approval | 24 | Plans for seeking research ethics committee/institutional review board (REC/IRB) approval | 24 | [M] Plans for seeking research ethics committee/institutional review board (REC/IRB) approval  WHO: include date of approval | Yes/ No |
|  | Protocol Amendments | 25 | Plans for communicating important protocol modifications (eg, changes to eligibility criteria, outcomes, analyses) to relevant parties (eg, investigators, REC/IRBs, trial participants, trial registries, journals, regulators) | 25 | Unchanged | Yes/ No |
|  | Consent or assent | 26a | Who will obtain informed consent or assent from potential trial participants or authorised surrogates, and how | 26a | Unchanged | Yes/ No |
|  |  | 26b | Additional consent provisions for collection and use of participant data and biological specimens in ancillary studies, if applicable. | 26b | Unchanged | Yes/ No/ Not applicable |
|  | Confidentiality | 27 | How personal information about potential and enrolled participants will be collected, shared, and maintained in order to protect confidentiality before, during, and after the trial. | 27 | Unchanged | Yes/ No |
|  | Declaration of Interest | 28 | Financial and other competing interests for principal investigators for the overall trial and each study site. | 28 | Unchanged | Yes/ No |
|  | Access to data | 29 | Statement of who will have access to the final trial dataset, and disclosure of contractual agreements that limit such access for investigators | 29 | Unchanged | Yes/ No |
|  | Ancillary and post-trial care | 30 | Provisions, if any, for ancillary and post-trial care, and for compensation to those who suffer harm from trial participation | 30 | Unchanged | Yes/ No |
|  | Dissemination policy | 31a | Trial results – Plans for investigators and sponsor to communicate trial results to participants, healthcare professionals, the public, and other relevant groups (eg, via publication, reporting in results databases, or other data sharing arrangements), including any publication restrictions | 31a.1 | Unchanged | Yes/ No |
|  |  |  |  | 31a.2 | Specify if results (e.g. DLT, response outcomes) can be shared whilst the trial is still ongoing. | Yes/ No/ Not applicable |
|  |  | 31b | Authorship – Authorship eligibility guidelines and any intended use of professional writers | 31b | Unchanged | Yes/ No |
|  |  | 31c | Reproducible research – Plans, if any, for granting public access to the full protocol, participant-level dataset, and statistical code | 31c | Unchanged | Yes/ No/ Not applicable |
| Appendices | Informed consent material | 32 | Model consent form and other related documentation given to participants and authorised surrogates. | 32 | Unchanged | Yes/ No |
|  | Biological specimen | 33 | Plans for collection, laboratory evaluation, and storage of biological specimens for genetic or molecular analysis in the current trial and for future use in ancillary studies, if applicable | 33 | Unchanged | Yes/ No/ Not applicable |
|  | Others |  |  | 34 | [N] Provide dose transition pathways or dose decision paths | Yes/No/ Not applicable |

## **Supplementary Table 2**: Percentage of protocols that adequately reported each item

| **Data Items** | **Review Item No.** | **SPIRIT 2013 Item Description** | **Percentage of protocols with adequately reported item***  **(%)** |
| --- | --- | --- | --- |
| Title | 1 | Descriptive title identifying the study design (e.g. first-in-human, early phase dose-finding, dose escalation/de-escalation, single-/multiple-ascending dose, phase I, (seamless) phase I/II or dose-titration), population, interventions, and, if applicable, randomisation and/or trial acronym | **78.3** |
| Trial Registration | 2 | Registry – Trial identifier and registry name. If not yet registered, name of intended registry. Data set – All items from the World Health Organization Trial Registration Data Set | **79.1** |
| Protocol Version | 3 | Date and version identifier | **98.1** |
| Funding | 4 | Sources and types of financial, material, and other support | **44.3** |
| Roles And Responsibilities | 5a | Contributorship - Names, affiliations, and roles of protocol contributors | **36.8** |
|  | 5b | Sponsor contact information – Name and contact information for the trial sponsor | **77.4** |
|  | 5c | Sponsor and funder – Role of study sponsor and funders, if any, in study design; collection, management, analysis, and interpretation of data; writing of the report; and the decision to submit the report for publication, including whether they will have ultimate authority over any of these activities | **66.0** |
|  | 5d | Committees - Composition, roles, and responsibilities of the coordinating centre, steering committee, endpoint adjudication committee, data management team, and other individuals or groups overseeing the trial, if applicable | **68.6** |
| Background And Rationale | 6a.1 | Description of research question and justification for undertaking the trial, including: summary of findings from nonclinical (e.g. in vitro, in vivo and/or in silico studies) that have potential clinical significance | **97.2** |
|  | 6a.2 | If applicable, summary of pre-clinical/non-clinical research | **93.2** |
|  | 6b | Explanation for choice of comparators | **47.1** |
| Objectives | 7 | Specific objectives or hypotheses; defining clearly what the key objectives are (e.g., primary and secondary objectives that encompasses: safety, toxicity, activity/efficacy, pharmacokinetics, pharmacodynamics, feasibility assessment, or some combination) | **100** |
| Trial Design | 8a | Description of trial design including type of trial (e.g., parallel group, crossover, factorial, single group), allocation ratio, and framework (e.g., superiority, equivalence, non-inferiority, exploratory) | **99.1** |
|  | 8b | Is the trial design schema provided? | **67.9** |
|  | 8c.1 | Where applicable, details regarding the statistical methodology underpinning the trial, including the choice of the number of parameters in the model if applicable, its empirical form and all formulae | **70.8** |
|  | 8c.2 | Rationale for starting dose | **68.9** |
|  | 8d | Planned cohort size(s) (fixed or flexible) | **100** |
|  | 8e | Sequence and interval between dosing of participants (e.g. sentinel/staggered dosing at each untested dose level or between dose levels) | **46.2** |
|  | 8f | Pre-planned guidance/rules for trial adaptations (e.g. dose [de-]escalation strategy), when they will occur and the information (e.g. toxicity, response, PK/PD, either singularly or in combination) used to make the adaptations | **74.3** |
|  | 8g | Stopping criteria for treatment group or trial (e.g. progression to the next part of the trial, early termination of a group or the trial due to e.g. safety, futility or efficacy) and consequences | **90.6** |
| Study Setting | 9 | Description of study settings (e.g., community clinic, academic hospital) and list of countries where data will be collected. Reference to where list of study sites can be obtained | **36.8** |
| Eligibility Criteria | 10 | Inclusion and exclusion criteria for participants. If applicable, eligibility criteria for study centres and individuals who will perform the interventions (e.g., surgeons, psychotherapists) | **100** |
| Interventions | 11a | The interventions for each dose level within each treatment group with sufficient details to allow replication, including administration, route, and schedule showing how and when they will be administered. | **97.2** |
|  | 11b | Criteria for discontinuing, dose modifications and dosing delays of allocated interventions for a given trial participant (e.g. drug dose change in response to harms, participant request, or improving /worsening disease) | **85.7** |
|  | 11c | Strategies to improve adherence to intervention protocols, and any procedures for monitoring adherence (e.g., drug tablet return; laboratory tests) | **45.5** |
|  | 11d | Relevant concomitant care and interventions that are permitted or prohibited during the trial | **92.5** |
| Outcomes | 12 | Primary, secondary and other outcome measures, including the specific measurement variable (eg, dose limiting toxicity based on drug-related adverse events), analysis metric (eg, change from baseline, final value, time to event), method of aggregation (eg, median, proportion), and time point for each outcome. Explanation of the clinical relevance of chosen efficacy and harm outcomes | **99.1** |
| Participant Timeline | 13 | Time schedule of enrolment, interventions (including any run-ins and washouts), assessments, and visits for participants (including in-house stay or out-patient follow-up period where applicable). A schematic diagram is highly recommended | **96.2** |
| Sample Size | 14a.1 | Estimated number of participants (minimum/lower bound, maximum or expected range) needed to address study objectives and how it was determined, including clinical and statistical assumptions | **86.8** |
|  | 14a.2 | Were simulations/operating characteristics provided? | **15.4** |
| Recruitment | 15 | Strategies for achieving adequate participant enrolment to reach target sample size. Method of recruitment (e.g., referral, self-selection), including the sampling method if a systematic sampling plan to be implemented | **30.2** |
| Allocation | 16 | Is the trial randomised? | **34.0** |
|  | 16a | Method of generating the allocation sequence (eg, computer-generated random numbers), and list of any factors for stratification. To reduce predictability of a random sequence, details of any planned restriction (eg, blocking) should be provided in a separate document that is unavailable to those who enrol participants or assign interventions | **68.6** |
|  | 16b | Concealment mechanism: Mechanism of implementing the allocation sequence (eg, central telephone; sequentially numbered, opaque, sealed envelopes), describing any steps to conceal the sequence until interventions are assigned | **50.0** |
|  | 16c | Logistics: Who will generate the allocation sequence, who will enrol participants, and who will assign participants to interventions. Inclusion of aspects employed to help minimize potential bias induced due to non-randomization if applicable (e.g., matching) | **54.3** |
| Blinding (Masking) | 17a | Who will be blinded after assignment to interventions (eg, trial participants, care providers, outcome assessors, data analysts), and how | **97.0** |
|  | 17b | If blinded, circumstances under which unblinding is permissible, and procedure for revealing a participant’s allocated intervention during the trial | **75.0** |
| Data Collection Methods | 18a.1 | Plans for assessment and collection of outcome, baseline, and other trial data, including any related processes to promote data quality (eg, duplicate measurements, training of assessors) and a description of study instruments (eg, questionnaires, laboratory tests) along with their reliability and validity, if known | **77.4** |
|  | 18a.2 | Reference to where data collection forms can be found, if not in the protocol | **32.4** |
|  | 18b | Plans to promote participant retention and complete follow-up, including list of any outcome data to be collected for participants who discontinue or deviate from intervention protocols | **45.3** |
| Data Management | 19a.1 | Plans for data entry, coding, security, and storage, including any related processes to promote data quality (eg, double data entry; range checks for data values) | **81.0** |
|  | 19a.2 | Reference to where details of data management procedures can be found, if not in the protocol. Where applicable, specify if the plans in the initial dose-finding component are different from subsequent stages of the trial | **40.9** |
| Statistical methods | 20a.1 | Statistical methods used for primary and secondary outcomes, and any other outcomes used to make pre-planned adaptations (e.g., determining the next participant’s dose level). | **89.6** |
|  | 20a.2 | Reference to where other details of the statistical analysis plan can be found, if not in the protocol. | **47.3** |
|  | 20b | Methods for any additional analyses (e.g. subgroup and adjusted analyses, PK/PD, biomarker correlative analyses) or where they can be found, if not in the protocol | **77.0** |
|  | 20c.1 | Definition of dose-escalation analysis population | **33.7** |
|  | 20c.2 | Definition of safety population | **66.0** |
|  | 20c.3 | Definition of other key outcomes population | **62.3** |
|  | 20c.4 | Statistical methods to handle missing data | **33.0** |
| Data monitoring | 21a | Composition of any decision-making group or safety review committee or data (safety) monitoring committee (DMC); summary of its role and reporting structure; statement of whether it is independent from the sponsor, funder or trials team and competing interests; and reference to where further details about its charter can be found, if not in the protocol. Alternatively, an explanation of why such a committee is not needed. | **81.1** |
|  | 21b | Interim analysis – Description of any interim analyses and stopping guidelines, including who will have access to these interim results and make the final decision to terminate the trial. | **71.7** |
| Harms | 22 | Plans for collecting, assessing, reporting, and managing solicited and spontaneously reported adverse events and other unintended effects of trial interventions (e.g. prior to any planned next dosing) or trial conduct | **96.2** |
| Auditing | 23 | Frequency and procedures for auditing trial conduct (including specifying any difference moving from e.g. FIH/phase I to dose expansion or phase II), if any, and whether the process will be independent from investigators and the sponsor. | **69.5** |
| Research Ethics Approval | 24 | Plans for seeking research ethics committee/institutional review board (REC/IRB) approval | **93.4** |
| Protocol Amendments | 25 | Plans for communicating important protocol modifications (eg, changes to eligibility criteria, outcomes, analyses) to relevant parties (eg, investigators, REC/IRBs, trial participants, trial registries, journals, regulators) | **79.1** |
| Consent or assent | 26a | Who will obtain informed consent or assent from potential trial participants or authorised surrogates, and how | **84.9** |
|  | 26b | Additional consent provisions for collection and use of participant data and biological specimens in ancillary studies, if applicable. | **47.9** |
| Confidentiality | 27 | How personal information about potential and enrolled participants will be collected, shared, and maintained in order to protect confidentiality before, during, and after the trial | **82.1** |
| Declaration of Interest | 28 | Financial and other competing interests for principal investigators for the overall trial and each study site. | **33.0** |
| Access to data | 29 | Statement of who will have access to the final trial dataset, and disclosure of contractual agreements that limit such access for investigators | **52.4** |
| Ancillary and post-trial care | 30 | Provisions, if any, for ancillary and post-trial care, and for compensation to those who suffer harm from trial participation | **26.7** |
| Dissemination policy | 31a.1 | Trial results – Plans for investigators and sponsor to communicate trial results to participants, healthcare professionals, the public, and other relevant groups (eg, via publication, reporting in results databases, or other data sharing arrangements), including any publication restrictions | **76.4** |
|  | 31a.2 | Specify if results (e.g. DLT, response outcomes) can be shared whilst the trial is still ongoing | **12.0** |
|  | 31b | Authorship – Authorship eligibility guidelines and any intended use of professional writers | **25.5** |
|  | 31c | Reproducible research – Plans, if any, for granting public access to the full protocol, participant-level dataset, and statistical code | **17.1** |
| Informed consent material | 32 | Model consent form and other related documentation given to participants and authorised surrogates. | **9.4** |
| Biological specimen | 33 | Plans for collection, laboratory evaluation, and storage of biological specimens for genetic or molecular analysis in the current trial and for future use in ancillary studies, if applicable | **56.8** |
| Others | 34 | Provide dose transition pathways or dose decision paths | **23.1** |

* Items evaluated as “*Yes*” or “*Partially*” were combined as ‘*adequately reported*’

# **Supplementary Figures**

## **Supplementary Figure 1**: Overview of the assessment strategy


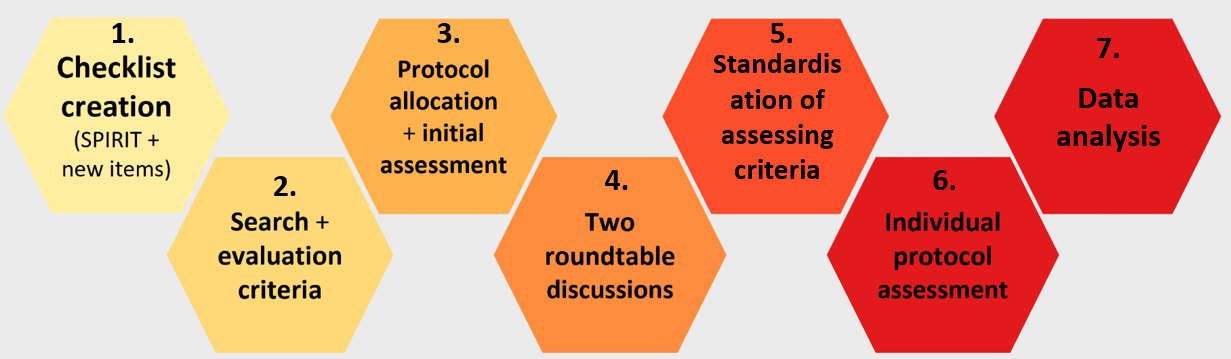


# **Supplementary Examples Information**

## **Illustrative examples of adequately and partially reported items in the evaluated dose-finding trial protocols**

| **Item 4:** Funding; sources and types of financial, material, and other support. | |
| --- | --- |
| **Adequately reported** | **Partially reported** |
| The study is financed by Deutsche Krebshilfe, grant 111004 to Univ.-Prof. Dr. Wolfgang E. Berdel. | Financing and Insurance are discussed in detail in the Clinical Trial Agreement. |
| **Item 7:** Specific objectives or hypotheses; defining clearly what the key objectives are (e.g., primary and secondary objectives that encompasses: safety, toxicity, activity/efficacy, pharmacokinetics, pharmacodynamics, feasibility assessment, or some combination) | |
| **Adequately reported** | **Partially reported** |
| Primary Objectives  Safety: To evaluate the safety and tolerability of the trivalent P2-VP8 subunit rotavirus vaccine at escalating dose levels in healthy South African adults, toddlers and infants  Immunogenicity: To evaluate the immunogenicity of three doses of the trivalent P2-VP8 subunit rotavirus vaccine at different dose levels in healthy South African infants.  Secondary Objectives  Safety: To evaluate the longer term safety (through 6 months after the last vaccination) of the trivalent P2-VP8 subunit rotavirus vaccine at escalating dose levels in healthy South African adults, toddlers and infants  Immunogenicity: To evaluate the immunogenicity of two doses of the trivalent P2-VP8 subunit rotavirus vaccine at different dose levels in healthy South African infants.  Exploratory Objective  Efficacy: To evaluate the impact of the trivalent P2-VP8 subunit rotavirus vaccination on shedding of Rotarix subsequently administered in healthy South African infants as a test of concept | The primary trial objective is to determine the MTD of nintedanib twice daily (b.i.d.) plus  weekly docetaxel 35 mg/m2 (day 1, day 8, day 15 of a 28-day cycle) in patients with locally  advanced or metastatic lung adenocarcinoma after failure of platinum-based first line  chemotherapy.  The further objectives concern the safety of nintedanib at each dose level in association with  weekly docetaxel, as well as the PK data of nintedanib when administered continuously with  docetaxel treatment. |
| **Item 8:** Trial design; Description of type and design of trial: i) Phase, ii) Type of trial (e.g. open-label, double-blinded, placebo-controlled, dose-escalation, expansion cohort, intra-patient dose escalation), iii) Framework (if applicable, Bayesian or frequentist hypothesis testing), iv) Number of study groups/arms with rationale. | |
| **Adequately reported** | **Partially reported** |
| The trial is a double-blind, randomized, placebo-controlled dose-escalation study in which two dose-levels (30 μg and 90 μg) of vaccine will be tested in adults and toddlers and then three dose-levels will be assessed in infants (15 μg, 30 μg and 90 μg). In Group A, cohorts of 15 adults (12 vaccine and 3 placebo recipients) per dose level will receive three study injections at 4 week intervals (consistent with the regimen in the adult study of the monovalent vaccine), advancing from the first to the second dose-level after assessment of safety data through the first week after the first injection (A1). Based on the safety data in the adult group, Group B cohorts of 15 toddlers (12 vaccine recipients and 3 placebo recipients) per dose level will receive a single intramuscular injection, etc… | This is a single-center Phase I/Ib study aiming to evaluate the safety, efficacy, and  tolerability of nivolumab combined with therasphere, Y-90, in patients with advanced  hepatocellular carcinoma. |

## **Handbook with examples of adequately reported items**

Administrative information

## Title

### Item 1:

Descriptive title identifying the study design (e.g. first-in-human, early phase dose-finding, dose escalation/de-escalation, single-/multiple-ascending dose, phase I, (seamless) phase I/II or dose-titration), population, interventions, and, if applicable, randomisation and/or trial acronym

Examples:

1. “Safety of sildenafil in premature infants with severe bronchopulmonary dysplasia (SILDI-SAFE): a multicentre, randomized, placebo-controlled, sequential dose escalating, double-masked, safety study.” [1]
2. “Phase I/II dose-escalation trial of combination fractionated-dose ^177^Lu-J591 and ^177^Lu-PSMA-617 in patients with metastatic castration-resistant prostate cancer.” [3]
3. “A First-in-Human, Randomized, Double-blind, Dose Escalation Study to Assess the Safety, Tolerability, Pharmacokinetics, and Pharmacodynamics Following Single and Multiple Oral Doses of KM-819 in Healthy Young Adult and Elderly Subjects.” [4]

## Trial registration

### Item 2:

Trial identifier and registry name. If not yet registered, name of intended registry

Example: “NCT04447989” [1]

## Protocol version

### Item 3:

Date and version identifier

Example:

“This protocol (Version Number 2.0; September 4, 2020)

Version Date: February 19, 2019 [3]

Date of Protocol: 13 Feb 2017, Final 5.0” [4]

## Roles and responsibilities

### Item 5a:

Names, affiliations, and roles of protocol contributors

Example:

“Authors’ contributions

SS, TS, CE, ML, CH, and WJ made substantial contributions to the conception and design of the study. SS and WJ wrote the manuscript, and TS, CE, MB, ML, and CH made edits. All authors have read and approved the final manuscript.” [1]

### Item 5b:

Name and contact information for the trial sponsor

Examples:

1.
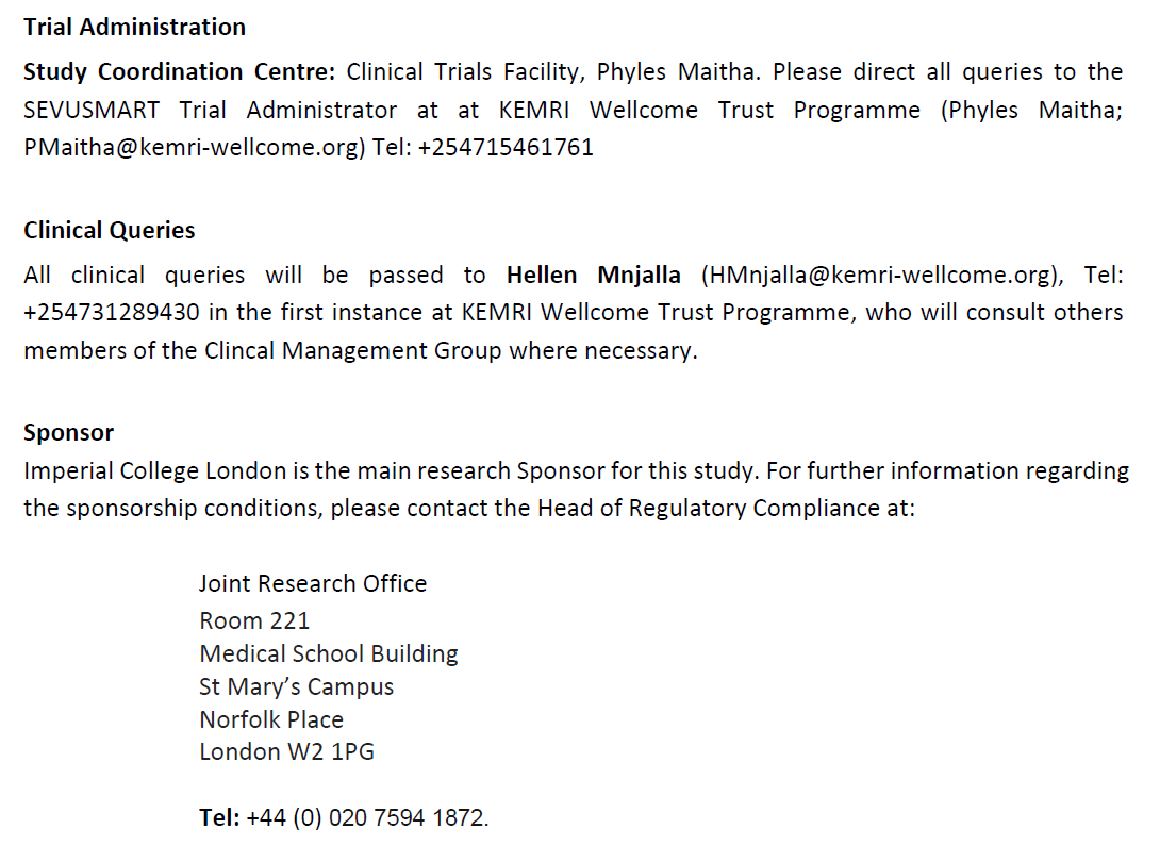
[2]
2.
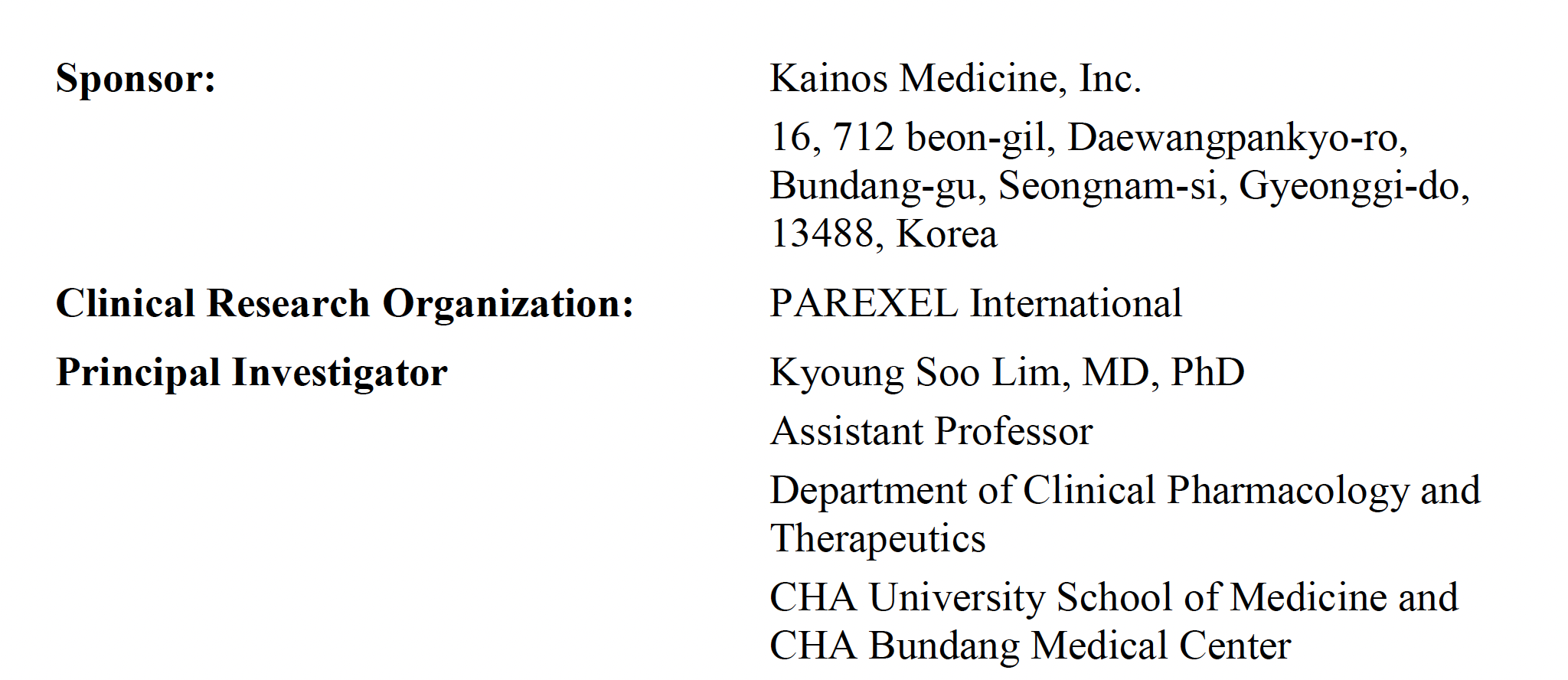
[4]

### Item 5c:

Role of study sponsor and funders, if any, in study design; collection, management, analysis, and interpretation of data; writing of the report; and the decision to submit the report for publication, including whether they will have ultimate authority over any of these activities

Example:


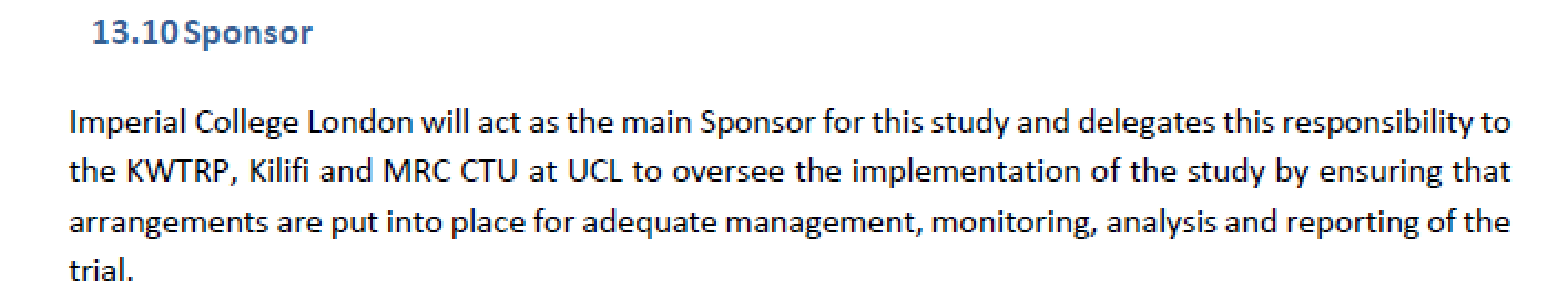
[2]

Introduction

## Background and rationale

## Item 6a.1:

Description of research question and justification for undertaking the trial, including: summary of findings from nonclinical (e.g. in vitro, in vivo and/or in silico studies) that have potential clinical significance

Examples:

1. “Modifications of PSMA−11 have resulted in the development of another novel small molecule PSMA−ligand, PSMA−617. In preclinical studies, this ligand showed a significantly improved binding affinity to PSMA as well as a highly efficient internalization into PCa cells (69). PSMA−617 can be labeled with radionuclides like 68Ga (Gallium−68), 177Lu(Lutetium−177), 111In(Indium−111), and 90Y(Yetrium−90) and, therefore, be used for PET−imaging as well as for radioligand−based therapy. Preclinical assays of PSMA−617 showed Ki values of 2.3 ± 2.9 nM, demonstrating a significant improvement compared to PSMA−11 (12.0 ± 2.8 nM). Based on these results, PSMA−617 has one of the highest binding affinities to the PSMA receptor, which have been published, so far. In preclinical studies, tumor−to−background ratios of up to 1,058 were observed at 24h post infusion. In addition, the internalization of the PSMA−617 into the PCa cells is highly effective: Internalized fraction: 17.67 ± 4.34 % IA/106 LNCaP cells (PSMA−11: 9.47 ± 2.56 % IA/106 LNCaP cells)(59, 69). Since 2013, 177Lu−PSMA−617 has been increasingly used for radioligand therapy of metastatic PCa patients in several centers (Bad Homburg, Bonn, Cologne, Freiburg, Heidelberg, Istanbul, Melbourne, LMU Munich, Münster) Preclinical data have shown that dose fractionation or multiple low dose treatments can decrease toxicity while increasing the efficacy.” [3]
2. “KM-819 is a drug candidate with EC50 value of nM range for in vitro cell death protection assay study (in human neuronal cell line 200.3 nM and rat primary neuron 134.5 nM), and confirmed to exhibit dopaminergic neuron cell protection in 1-methyl-4-phenyl-1,2,3,6-tetrahydropyridine (MPTP)-induced Parkinson’s disease animal models, and to have a superior neuron cell protection effects and the similar behavioral improvement effects compared to L-dopa. In addition, it demonstrates specific binding to the target protein FAF1 upon cell death related to Parkinson’s disease.” [4]

## Item 6a.2:

If applicable, summary of relevant clinical research (published and unpublished) and any history of human use or exposure to the intervention.

Examples:

1.
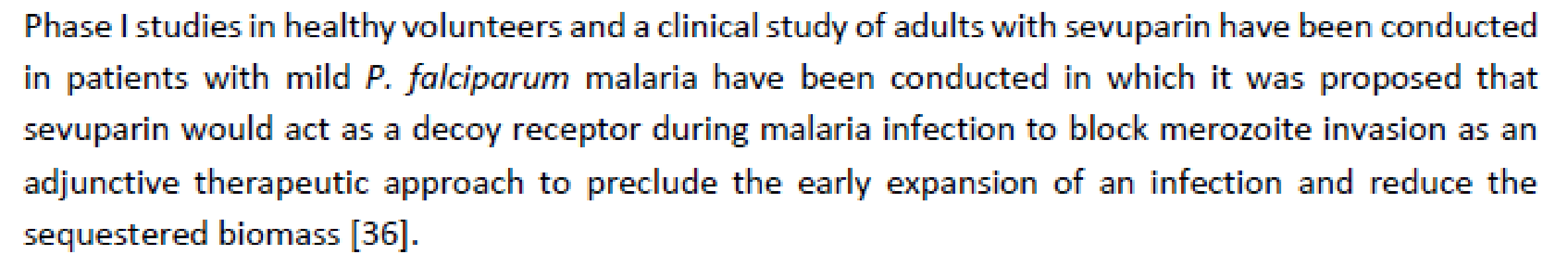
[2]
2. “In clinic, several orally bioavailable PARP inhibitors are used as standard of care or being developed. No clinical data on any PARP inhibitor in combination with radiotherapy was available at the time of the design of our combination trials.” [5]

## Item 6b:

Explanation for choice of comparators

Example:

“…the control group will consist of 24 patients who receive no drug but are followed while in-hospital (active controls). The control group will receive standard treatments guided by the institutional sepsis alert protocol.” [6]

## Objectives

### Item 7:

Specific objectives or hypotheses; defining clearly what the key objectives are (e.g., primary and secondary objectives that encompasses, safety, toxicity, activity/efficacy, pharmacokinetics, pharmacodynamics, feasibility assessment, or some combination)

Example:

“1. STUDY OBJECTIVES

The objectives of this clinical trial are as follows:

**1.1 Primary Objectives**

- (Phase I) Determine the dose limiting toxicity (DLT) of the combination of 177Lu−J591 and 177Lu−PSMA−617 in a 2−week dose−fractionation regimen
- (Phase I) Determine the cumulative maximum tolerated dose (MTD) and/or recommended phase II dose (RP2D) of the combination of 177Lu−J591 and 177Lu−PSMA−617 in a 2−week dose−fractionation regimen
- (Phase II) To assess the proportion with PSA decline following treatment with the combination of 177Lu−J591 and 177Lu−PSMA−617 in a 2−week dose−fractionation regimen

**1.2 Secondary Objectives**

- To assess radiographic response rate by RECIST 1.1 with PCWG3 modifications
- To assess biochemical and radiographic progression−free survival by PCWG3 criteria To assess overall survival following treatment with the combination of 177Lu−J591 and 177Lu−PSMA−617 in a 2−week dose−fractionation regimen
- To assess safety of treatment with the combination of 177Lu−J591 and 177Lu−PSMA−617 in a 2−week dose−fractionation regimen as assessed by CTCAE 4.0
- To assess changes in CTC count as measured by CellSearch and the rate of favorable CTC count and LDH at 12 weeks following treatment with the combination of 177Lu−J591 and 177Lu−PSMA−617 in a 2−week dose−fractionation regimen
- To assess patient reported outcomes using FACT−P and the Brief Pain Inventory short form

**1.3 Exploratory Objectives**

- Disease assessment with PSMA−ligand based imaging prior to and following investigational treatment
- To assess immune effects of PSMA−targeted radionuclide therapy
- To assess genomic alterations in relationship to outcome following treatment with the combination of 177Lu−J591 and 177Lu−PSMA−617 in a 2−week dose−fractionation regimen
- To estimate whole body distribution and radiation dosimetry of 177Lu−J591 and 177Lu−PSMA−617” [3]

## Trial design

### Item 8a:

Description of type and design of trial (e.g. open-label, double-blinded, placebo-controlled, dose-escalation, expansion cohort, intra-patient dose escalation, algorithm-based, model-based, Bayesian), including allocation ratio if relevant, and number of study groups/arms.

Examples:

1. “Phase I:

The dose-escalation schedule (using 3+3 modified Fibonacci escalation), definitions of DLT, and determination of MTD are defined above (Section 6.2.10). The design is constructed to reduce the chance of escalating the dose when the probability of DLT is high, and increase the chance of escalating the dose when the probability of DLT is low. The maximum tolerated dose is defined as the highest dose level with an observed incidence of DLT in no more than one out of six patients treated at a particular dose level. The dose escalation scheme provides the following probabilities of escalation based on the true chances of DLT at a specific dose level. One can see that the probability of escalation is high if the toxicity risks are low.

True Probability of Toxicity 0.05 0.10 0.20 0.30 0.40 0.50 0.60

Probability of Escalation 0.97 0.91 0.71 0.49 0.31 0.17 0.08” [3]

1. “This is a first-in-human, single-center, randomized, placebo-controlled, double-blind, sequential group Phase 1 study in healthy subjects. The aim of this study is to evaluate the safety, tolerability, PK, and PD following the escalation of single and multiple doses of KM-819. The study will consist of 2 parts (Part A and Part B).
   1. Part A includes up to 5 cohorts of healthy young adult male subjects receiving single ascending doses (SAD)s of KM-819, and 1 additional single-dose cohort of elderly male or post-menopausal female subjects.
   2. Part B includes up to 4 cohorts of healthy young adult male subjects receiving multiple ascending doses (MAD)s of KM-819, and 1 additional multiple-dose cohort of elderly male or post-menopausal female subjects.

Dose escalation to the next level will be determined using the safety, tolerability, and PK data of the previous cohort.” [4]

### New Item 8c.2:

Rationale for starting dose

Example:

“The initial dosage (1.5 mg/kg) and the *a priori* dose-toxicity curve are based upon the results of the adult trial (where a dose of 1.5 mg/kg was associated with minimal risk of toxicity) and experimental evidence of dose-dependent efficacy i.e. inhibition of merozoite invasion and reversal of cytoadherence of infected erythrocytes [36]. Almost all adults enrolled in this trial experienced grade 2 toxicity after one or more sevuparin doses, but APTT rapidly normalized, hence the choice of grade 3 toxicity to define the MTD in this dose-finding trial.” [2]

### New Item 8d:

Planned cohort size(s) (fixed or flexible), if applicable (see Item #14, sample size)

Examples:

1. “The initial participants (two ‘cohorts’ of 2 children each, i.e. 4 children in total) will receive a dose of 1.5 mg/kg/dose with the plan to escalate up to a cohort of 2 children receiving 3mg/kg/dose and a cohort of two children receiving 6.0 mg/kg/dose (maximum).” [2]
2. “Each of the 5 dose escalation cohorts consists of 8 healthy young adult male subjects; 6 subjects will receive single dose of 10, 30, 100, 200, or 400 mg KM-819 and 2 subjects will receive placebo.” [4]

### New Item 8e:

Sequence and interval between dosing of participants (e.g. sentinel/staggered dosing at each untested dose level or between dose levels).

Examples:

1. “In Part A, escalation to the next dose level will take place only after the safety and tolerability data for all subjects in the cohort (through Day 4) and available plasma PK data (through the 72-hour sample postdose) from the previously administered dose cohort has been reviewed. Data from exploratory PD assessments and CNS scales will not be included in the review for dose escalation decisions. Within the planned dose range a dose lower than the next planned dose level may be tested, depending on the emerging safety, tolerability, and/or other relevant data (e.g., plasma PK data). In each single dose cohort (Part A), dosing of subjects will be sentinel, i.e., 2 subjects will be dosed on the first day (1 subject will receive active treatment and 1 subject will receive placebo) and the remaining 6 subjects will be dosed at least 24 hours after the first 2 subjects.” [4]
2. “The first three patients in each trial arm are treated at the starting dose level. Thereafter, patients are assigned to a dose level using TITE-CRM and dose escalation rules. Upon enrolment of a new patient, TITE-CRM estimates the current MTD (see statistical analysis). New patients are assigned to the dose level that is closest to but not exceeding this current estimated MTD after applying two restrictive dose-escalation rules: 1) at least three patients have completed a minimal follow-up time of three months after end of treatment at the dose level below the assigned dose level, and 2) the assigned dose level may not increase more than one dose level between two consecutive patients. There is no restriction on the decrease in number of levels between consecutive patients.” [5]

### New Item 8g:

Stopping criteria (e.g. for individual participant, within a cohort, futility, progression to the next part of the trial or early termination of the trial) and consequences

Examples:

1. “The investigators or physicians may stop the protocol or terminate a subject’s participation in the protocol at any time should they judge:

- That it is not in the subject’s best interest to continue
- If the subject experiences a protocol–related injury
- If the subject needs life–saving medications/procedures/treatment
- If the subject does not comply with the study plan

General or specific changes in the patient’s condition render the patient unacceptable for further treatment in the judgment of the investigator” [3]

1. “Dosing will be stopped if 1 (or more) of the following apply. Depending on the nature of the AEs, it could be decided to investigate a lower dose level (intermediate between the current and the prior one) in the next group.
   1. If 1 or more subjects experiences a study drug-related serious adverse event (SAE), unblinding of the subject(s) will be done. If the subject(s) was (were) on active treatment, dosing will be stopped.
   2. If 2 subjects in 1 dose group show the following findings in 2 consecutive postdose measurements within 24 hours and if unblinding reveals that both subjects received active treatment, dosing will be stopped: a. ALT or AST ≥ 3 x upper limit of normal (ULN) or,
   3. ALT or AST ≥ 2 x ULN and ALT or AST ≥ 5 x baseline value or,
   4. TBIL ≥ 2 x ULN

If 2 or more subjects in 1 cohort experience AEs of severe intensity or 4 or more subjects in 1 cohort experience AEs of moderate intensity, which are considered by the PI and/or Sponsor to be possibly or probably related to the investigational product, and of clinical concern, and if unblinding reveals that these subjects received active treatment, dosing will be stopped. And:

If predicted mean AUC and Cmax for the next dose level is higher than 41.3 ug.h/mL and 9.3 ug/mL, respectively, then the dose escalation will stop and the Sponsor and PI will need to review the totality of data, including safety and PK before make any dose escalation decision. This PK stopping criteria are based on dog 2-Week study NOAEL exposure at 1000 mg/kg/day (the most conservative species and gender).” [4]

Methods: Participants, interventions, and outcomes

## Study setting

### Item 9:

Description of study settings (eg, community clinic, academic hospital) and list of countries where data will be collected. Reference to where list of study sites can be obtained

Examples:

1. “High dependency ward in Kilifi County Hospital, Kenya.” [2]
2. “Participating Sites: Weill Cornell Medicine, Dana Farber Cancer Institute, Tulane Cancer Center” [3]

## Interventions

### Item 11a:

The interventions for each dose level within each treatment group with sufficient details to allow replication, including administration, route, and schedule showing how and when they will be administered:

For every planned dose:

- The form (e.g. tablet, intravenously, single injection, etc.)
- Time (e.g. morning/afternoon/evening, daily/weekly, before/during/after meal, etc.)
- Duration (e.g. 12 weeks, a year) and maintenance dosing
- Setting of drug administration (e.g. take the drug at home, primary/secondary/tertiary care centre, etc.) which is not the same as item #9.

### Item 11c:

Strategies to improve adherence to intervention protocols, and any procedures for monitoring adherence (eg, drug tablet return, laboratory tests) activities to increase compliance or adherence (e.g., incentives)

Example:


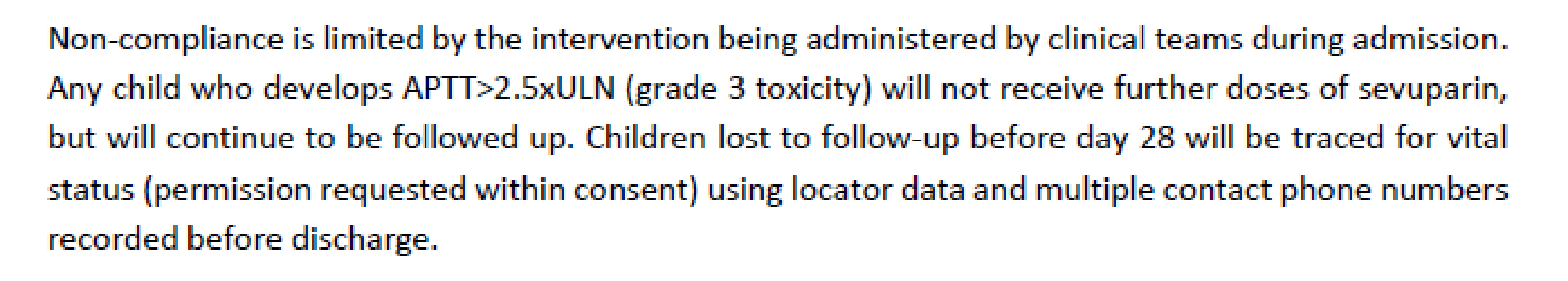
[2]

### Item 11d:

Relevant concomitant care and interventions that are permitted or prohibited during the trial

Example:


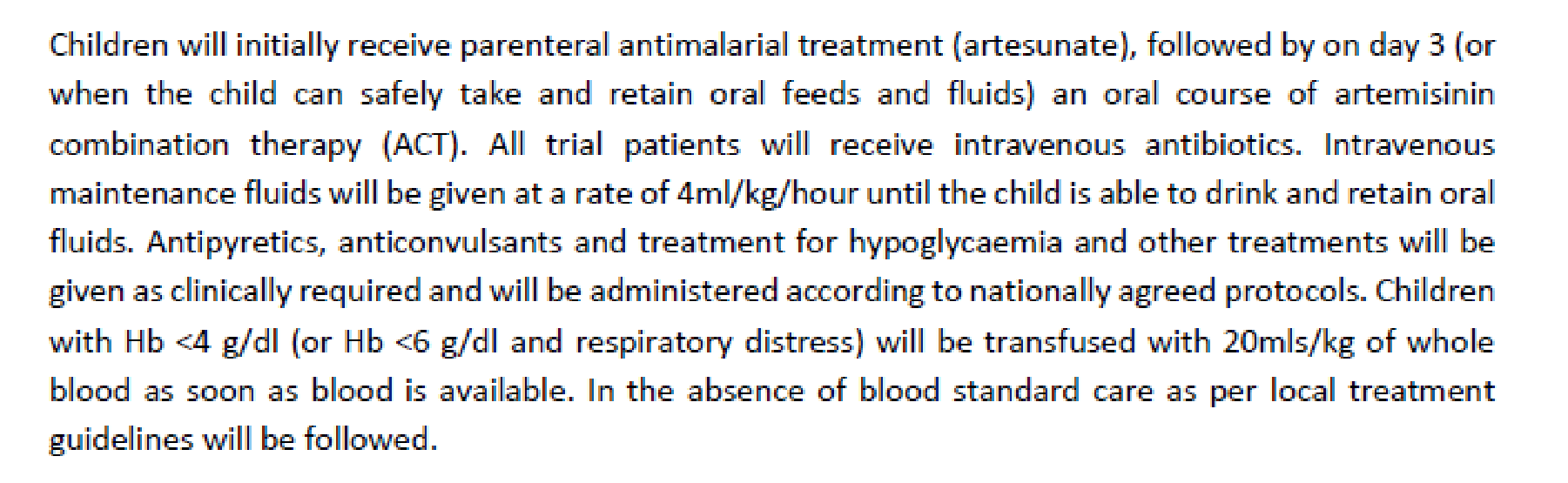
[2]

## Participant timeline

### Item 13:

Time schedule of enrolment, interventions (including any run-ins and washouts), assessments, and visits for participants (including in-house stay or out-patient follow-up period where applicable). A schematic diagram is highly recommended

Examples:


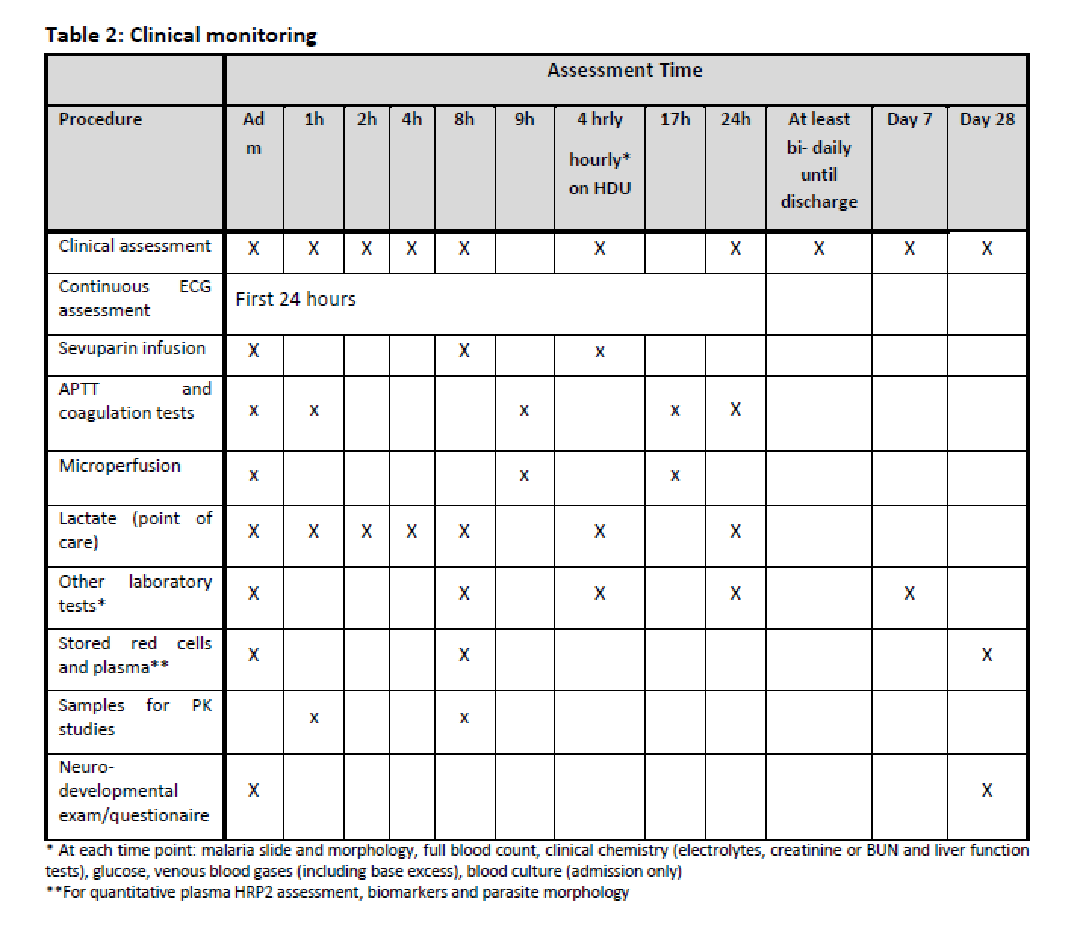
[2]


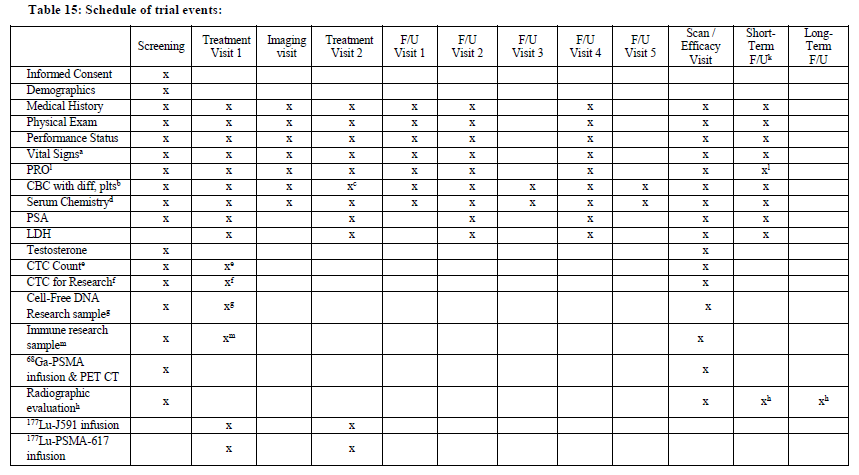
[3]

## Sample size

### Item 14:

Estimated number of participants needed to achieve study objectives and how it was determined, including clinical and statistical assumptions

Examples:

1. “We aim to study 20 children since this will allow sufficient data on safety to be generated across a range of doses to identify the maximum tolerated dose (MTD) from a more informed model relating dose to toxicity events (denoted the ‘dose-toxicity’ curve) than that available a priori based on published data from adult studies. After each patient is enrolled, the dose-toxicity curve will be updated based on levels of APTT taken over three time points (1h post each infusion), defining a toxicity event as APTT >2.5xULN at any time point (grade 3 following the Common Toxicity Criteria (CTC)). This enables the MTD to be estimated more rapidly using the Continuous Reassessment Method (CRM): once determined, subsequent participants will be allocated to this MTD to provide the most accurate estimate of future toxicity event rates until we reach the sample size of 20 children. However, the CRM method will continue to use information from all these children; for example, if a number of children receiving the originally identified MTD experience toxicity events, the dose would again be lowered, and future children would receive this lower dose.” [2]
2. “Sample size recommendations for the two-stage design are determined according to Simon’s two-stage minimax design. We project a 30% PSA decline proportion of 35%, below which the regimen will be unacceptable and a 30% PSA decline proportion of 60%, above which the regimen will be considered worthy of further exploration. The null hypothesis that the 30% PSA decline proportion is less than or equal to 35% will be tested against the alternative hypothesis that the 30% PSA decline proportion is greater than or equal to 60%.

The sample size computations were performed assuming a 0.10 one-sided level of significance and 90% power assuming a historical control value for PSA decline of 35% and the alternative hypothesis of 60%. If 6 or fewer of the first 16 evaluable patients do not experience a 30% decline in PSA (stage 1), the study will be terminated and declared to have a negative result. If 7 or more patients out of the first 16 evaluable patients experience a 30% decline in PSA, ongoing accrual will proceed to the target sample size of 27 patients (stage 2). The new regimen will be declared active in this patient population and worthy of further testing if 13 or more patients experience a 30% PSA decline among the 27 patients entered. This two-stage design yields a 0.90 probability of a positive result if the true 30% PSA decline proportion is 60%. It yields a 0.90 probability of a negative result if the true 30% PSA decline proportion is 35%. An exact 95% binomial confidence interval will be constructed for the proportion of patients with PSA decline.

12.3 Sample Size/Accrual Rate

The planned sample size for this dose escalation Phase I study is between 6 and 24 total patients and the accrual for phase II will be between 16 and 27 patients and will include patients treated at the MTD from the phase I portion. The accrual rate is anticipated to be 1−2 subjects every month on average (with expected pauses in enrollment between dose-escalation cohorts and between the stage 1 and stage 2 of the phase II trial).” [3]

## Recruitment

### Item 15:

Strategies for achieving adequate participant enrolment to reach target sample size + Method of recruitment (e.g., referral, self-selection), including the sampling method if a systematic sampling plan to be implemented

Example:

“Patients diagnosed with documented progressive metastatic CRPC disease who are visiting Oncology Clinic at NYPH−Cornell Campus for their standard of care visit, will be approached for recruitment for this study.” [3]

Methods: Data collection, management, and analysis

## Data management

### Item 19a.1:

Plans for data entry, coding, security, and storage, including any related processes to promote data quality (eg, double data entry; range checks for data values).

Examples:

“11.1.1. REDCap

REDCap (Research Electronic Data Capture) is a free data management software system that is fully supported by the Weill−Cornell Medical Center CTSC. It is a tool for the creation of customized, secure data management systems that include Web−based data−entry forms, reporting tools, and a full array of security features including user and group based privileges, authentication using institution LDAP system, with a full audit trail of data manipulation and export procedures.

REDCap is maintained on CTSC−owned servers that are backed up nightly and support encrypted (SSL−based) connections. Nationally, the software is developed, enhanced and supported through a multi−institutional consortium led by the Vanderbilt University CTSA.” [3]

Methods: Monitoring

## Data monitoring - formal committee

### Item 21a:

Composition of any decision making group or safety review committee or data (safety) monitoring committee (DMC); summary of its role and reporting structure; statement of whether it is independent from the sponsor, funder or trials team and competing interests; and reference to where further details about its charter can be found, if not in the protocol. Alternatively, an explanation of why such a committee is not needed.

Example:

“The Weill Cornell’s DSMB is comprised of medical specialists and advisors on human rights issues in human subjects research. The DSMB currently has 9 members, meets at quarterly intervals during the year, and carries out ongoing review of protocols submitted throughout the year. Once a protocol has been submitted and approved by the Institutional Review Board (IRB) and is recommended for oversight by the DSMB, the Board determines if the protocol will be reviewed quarterly, semi−annually, or annually.

The DSMB evaluates the accumulated data from the study in order to monitor the safety of subjects throughout the trial and reviews the risks and benefits, as well as the efficacy, of the study. The DSMB will also evaluate the overall trial conduct and progress. Ultimately, the DSMB validates the continuation of the trial or determines if a study needs modification or termination.

For this study, the DSMB will be notified after each cohort has been completed prior to dose escalation to the next cohort and prior to transition from phase I to phase II. In addition, a report will be made to the DSMB every 6 months.” [3]

## Harms

### Item 22:

Plans for collecting, assessing, reporting, and managing solicited and spontaneously reported adverse events and other unintended effects of trial interventions (e.g. prior to any planned next dosing) or trial conduct

Example:


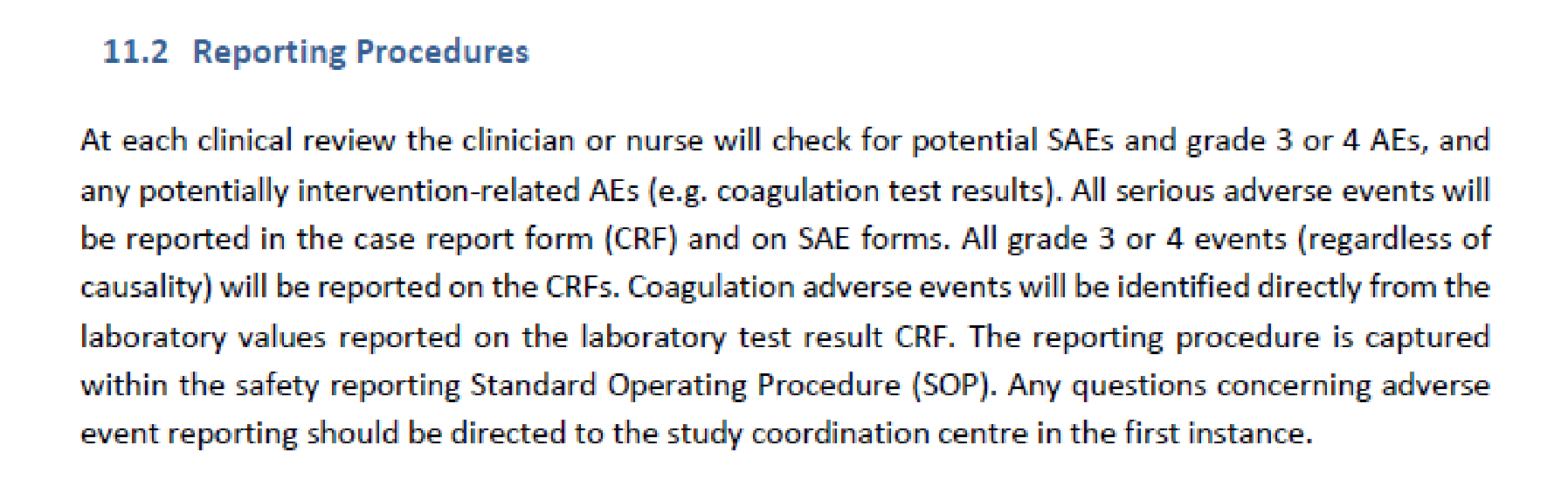
[3]

Ethics and dissemination

## Research ethics approval

### Item 24:

Plans for seeking research ethics committee/institutional review board (REC/IRB) approval

Examples:


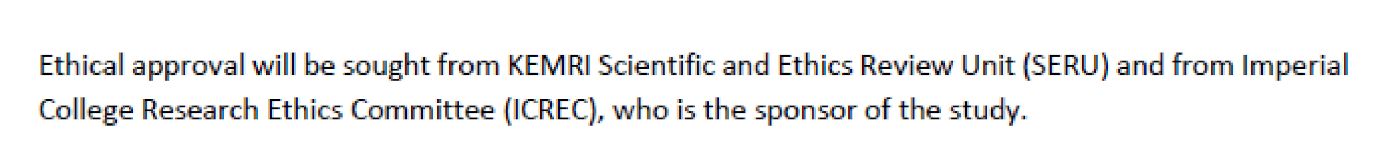
 [2]

## Consent or assent

### Item 26a:

Who will obtain informed consent or assent from potential trial participants or authorised surrogates, and how. If consent/assent is not obtained, why not (see Item 32).

Examples:

1. “Potential subjects will have a discussion with the investigator/delegate including the rationale for the study, investigational nature of the protocol and study drug and the voluntary nature of participation, potential risks and benefits, alternatives to participation, and study procedures. Individuals will have the opportunity to read the written informed consent document at their leisure (preferably outside of the clinical area for > 1 day) and the opportunity to have questions answered in a private location with the understanding that should they decide not to participate, they will still be able to receive any available standard of care therapy. Potential subjects will also have the opportunity to obtain the advice of their treating physician. Investigators or delegates under their direct supervision will verify the subject’s understanding of the investigational and voluntary nature of the study, the potential risks and benefits, study procedures, and alternatives prior to signing of the written informed consent.” [3]
2.
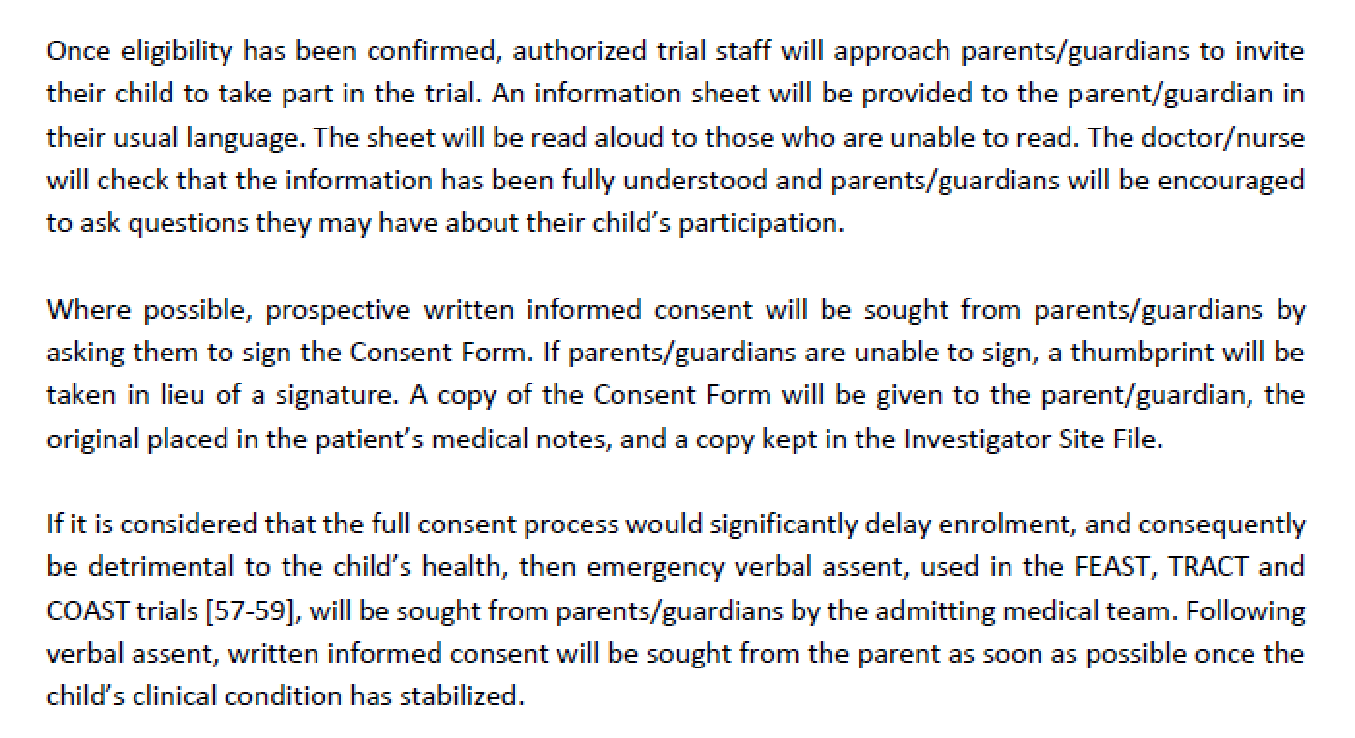
[2]

## Confidentiality

### Item 27:

How personal information about potential and enrolled participants will be collected, shared, and maintained in order to protect confidentiality before, during, and after the trial

Example:


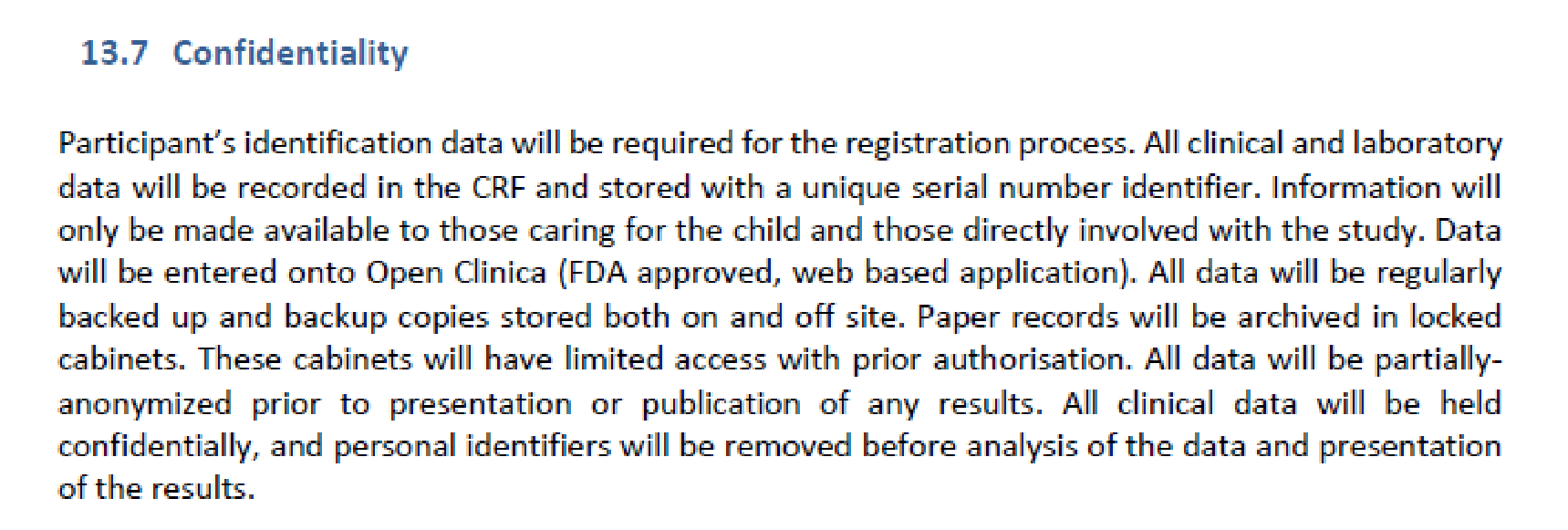
[2]

## Access to data

### Item 29:

Statement of who will have access to the final trial dataset, and disclosure of contractual agreements that limit such access for investigators

Examples:


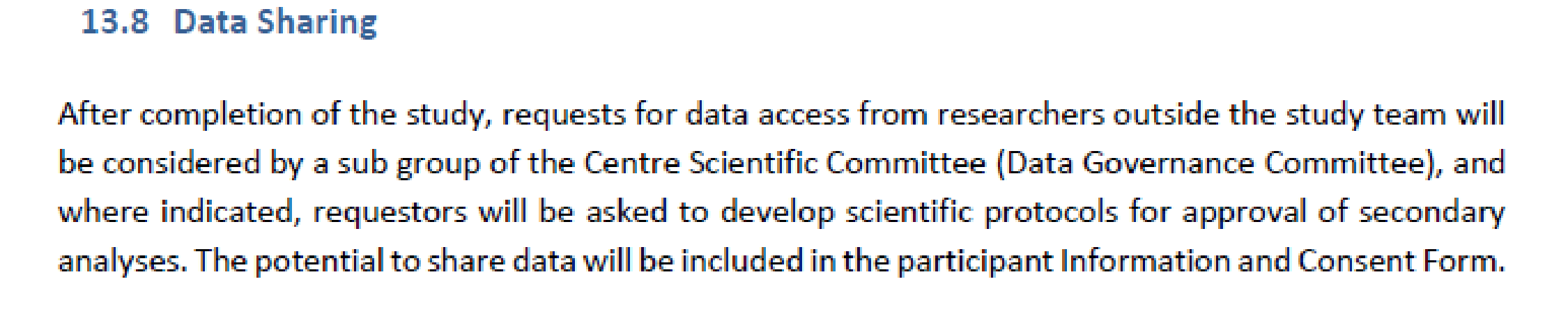
 [2]

Appendices

## Biological specimens

### Item 33:

Plans for collection, laboratory evaluation, and storage of biological specimens for genetic or molecular analysis in the current trial and for future use in ancillary studies, if applicable

Example:

“Archival tissue will be requested during the screening visit. Fifteen unstained slides containing tumor material from archival paraffin−embedded tissue should be obtained. If available, metastatic tissue is preferred to prostate biopsy/prostatectomy specimens. The tissue will be analyzed for PSMA expression and DNA damage repair pathways for research purposes only.” [3]

## Dose transition pathways (DTP)

### Item 34:

Provide dose transition pathways or dose decision paths

Example:


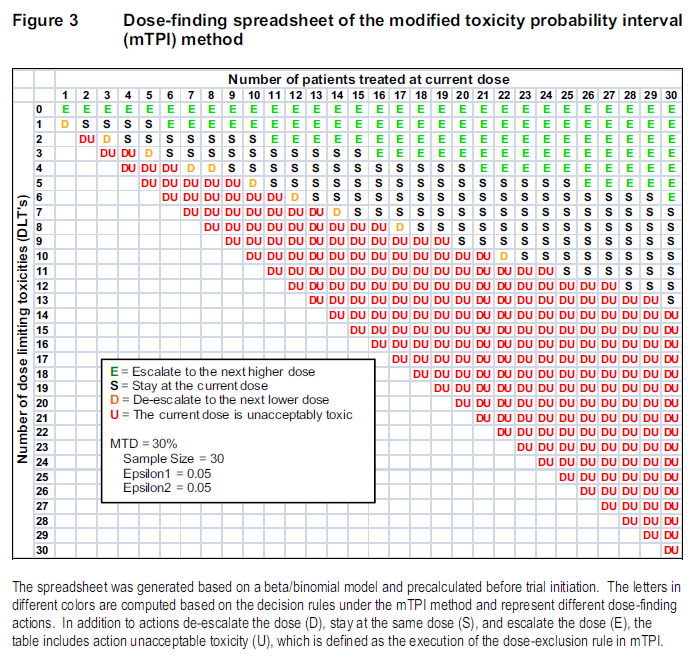
[7]

References:

1. A phase 1b, randomized, open-label study of pegylated recombinant human hyaluronidase (pegph20) in combination with cisplatin plus gemcitabine and pegph20 in combination with atezolizumab and cisplatin plus gemcitabine compared with cisplatin plus gemcitabine in subjects with previously untreated, unresectable, locally advanced, or metastatic intrahepatic and extrahepatic cholangiocarcinoma and gallbladder adenocarcinoma. NCT03267940.
2. Sevuparin as a potential adjunctive therapy in children with severe malaria: phase I safety and dose finding trial (SEVUSMART trial), 2020.
3. Phase I/II dose-escalation trial of combination fractionated-dose ^177^Lu-J591 and ^177^Lu-PSMA-617 in patients with metastatic castration-resistant prostate cancer, 2019. NCT03545165.
4. Shin W, Lim KS, Kim MK, Kim HS, Hong J, Jhee S, Kim J, Yoo S, Chung YT, Lee JM, Cho DY. A first-in-human study to investigate the safety, tolerability, pharmacokinetics, and pharmacodynamics of KM-819 (FAS-associated factor 1 inhibitor), a drug for Parkinson's disease, in healthy volunteers. Drug Des Devel Ther. 2019 Mar 29;13:1011-1022. doi: 10.2147/DDDT.S198753. PMID: 30992659; PMCID: PMC6445238.
5. de Haan R, van Werkhoven E, van den Heuvel MM, Peulen HMU, Sonke GS, Elkhuizen P, van den Brekel MWM, Tesselaar MET, Vens C, Schellens JHM, van Triest B, Verheij M. Study protocols of three parallel phase 1 trials combining radical radiotherapy with the PARP inhibitor olaparib. BMC Cancer. 2019 Sep 10;19(1):901. doi: 10.1186/s12885-019-6121-3.
6. Guirgis FW, Black LP, Rosenthal MD, Henson M, Ferreira J, Leeuwenburgh C, Kalynych C, Moldawer LL, Miller T, Jones L, Crandall M, Reddy ST, Wu SS, Moore FA. LIPid Intensive Drug therapy for Sepsis Pilot (LIPIDS-P): Phase I/II clinical trial protocol of lipid emulsion therapy for stabilising cholesterol levels in sepsis and septic shock. BMJ Open. 2019 Sep 18;9(9):e029348. doi: 10.1136/bmjopen-2019-029348. PMID: 31537565; PMCID: PMC6756323.
7. Dose Escalation and Expansion Study of GSK525762 in Combination With Fulvestrant in Participants With Hormone Receptor-positive (HR+)/Human Epidermal Growth Factor Receptor 2 Negative (HER2-) Advanced or Metastatic Breast Cancer. NCT02964507.
